# Distinct contributions of two subpopulations of subthalamic neurons to levodopa-induced dyskinesia

**DOI:** 10.1126/sciadv.aed2912

**Published:** 2026-07-03

**Authors:** Bo Shen, Linlin Han, Yan Xiao, Cong Shen, Ziwei Le, Bin Wu, Lu Su, Wenbo Yu, Yimin Sun, Fengtao Liu, Haishan Yao, Jianjun Wu, Jian Wang

**Affiliations:** ^1^ Department of Neurology and National Research Center for Aging and Medicine & National Center for Neurological Disorders, State Key Laboratory of Brain Function and Disorders, Huashan Hospital, Fudan University, Shanghai, China.; ^2^ Department of Geriatrics, Huashan Hospital, Fudan University, Shanghai, China.; ^3^ Department of Neurology, Xiangya Hospital, Central South University, Changsha, China.; ^4^ Institute of Neuroscience, State Key Laboratory of Neuroscience, CAS Center for Excellence in Brain Science and Intelligence Technology, Chinese Academy of Sciences, Shanghai, China.

## Abstract

The subthalamic nucleus (STN) is a prominent target for deep-brain stimulation (DBS) in the treatment of levodopa-induced dyskinesia (LID), a common motor complication of Parkinson’s disease. However, the precise impact of STN-DBS on LID remains unclear. Here, we investigated the functional roles of two distinct neuronal populations within the STN in regulating LID. In a mouse model of LID, STN neurons projecting to the entopeduncular nucleus (EP) exhibited a U-shaped activation pattern, whereas those projecting to the tegmental reticular nucleus (RtTg) displayed a predominantly inhibitory response. Activation of EP-projecting STN neurons alleviated dyskinesia but worsened hypokinesia in the parkinsonian state. Activation of RtTg-projecting STN neurons alone did not induce hyperkinetic characteristics, except when combined with levodopa. These findings reveal two anatomically and functionally distinct populations of STN neurons involved in LID regulation, offering insights into the circuitry underlying STN-DBS.

## Teaser

Distinct STN neuron populations differentially regulate LID via projections to EP and RtTg.

## INTRODUCTION

Levodopa remains the most effective medication for relieving motor symptoms in Parkinson’s disease (PD). However, long-term administration of levodopa often leads to the development of abnormal involuntary movements (AIMs), commonly referred to as levodopa-induced dyskinesia (LID). LID affects ~30% of patients with PD within the first 5 years of levodopa treatment and possibly 71% after 10 years of follow-up (*1*, *2*). Despite its clinical importance, the precise mechanisms responsible for the development of LID pathology remain largely elusive.

Although it has been shown that modulation of multiple brain regions can alleviate parkinsonian hypokinesia, only a few brain regions have been directly implicated in the initiation or maintenance of dyskinesia. The bidirectional regulation of locomotion by basal ganglia circuitry is well established (*3*), and imbalanced and paradoxical activity within the striatum—the input nucleus of the basal ganglia—have been observed during LID. Specifically, the direct pathway striatal spiny projection neurons (dSPNs) exhibit uncorrelated hyperactivity that correlates with the severity of dyskinetic behavior (*3*, *4*). On the other hand, hyperactivity in the indirect pathway striatal spiny projection neurons (iSPNs) is primarily hypothesized to occur during the parkinsonian state, and modulating the projection target of iSPNs has limited effects on locomotion (*5*). The subthalamic nucleus (STN) serves as a crucial node in the indirect pathway and a major target of clinical deep-brain stimulation (DBS) in PD (*6*, *7*). Despite its significance, the mechanisms by which the STN regulates the locomotor state remain contradictory and elusive.

The STN receives γ-aminobutyric acid-mediated (GABAergic) input from the external globus pallidus (GPe) and projects along the indirect pathway to regulate the inhibition of the internal globus pallidus (GPi) and the substantia nigra pars reticulata (SNr), as well as receives glutamatergic inputs from the cerebral cortex to form the hyperdirect pathway (*8*, *9*). Previous studies found that STN inhibition is sufficient to induce hyperkinesia, while STN activation reduces locomotion (*10*, *11*), suggesting that the effect of STN activity on locomotion is consistent with the rate model. It is worth noting that conflicting evidence exists from various studies, which involved pharmacological, electrophysiological, and optogenetic approaches in both rodent and nonhuman primate models, suggesting that stimulation of the STN may have the potential to exacerbate dyskinesia (*11*–*13*). This paradoxical phenomenon was indirectly supported by recent findings, which demonstrated the concurrent activation of the direct and indirect pathways during movement initiation (*4*, *14*). However, further investigation is required to understand the involvement of the STN in dyskinesia precisely.

STN-DBS is an effective and well-tolerated treatment of PD when it is difficult to balance the medication dose of levodopa between dyskinesia and parkinsonian symptoms. The specific stimulation site plays a pivotal role in the antiparkinsonian effects of STN-DBS, as the selection of a particular electrode contact can induce dyskinesia even in the absence of antiparkinsonian medication (*15*–*17*). Conventionally, the antidyskinesia effects of STN-DBS were attributed to a reduction in the levodopa equivalent daily dose (*18*, *19*). However, it has been shown that selecting the dorsal electrode within the STN can yield practical antidyskinesia effects (*20*). Clinical studies involving patients with PD without medication reduction have confirmed the direct suppressive effect of STN-DBS on LID (*21*). These intriguing yet contradictory findings suggest the potential existence of distinct subsets of STN neurons with heterogeneous functions.

In this study, we identified two anatomically distinct subsets of STN neurons on the basis of their projections to entopeduncular nucleus (EP) and tegmental reticular nucleus (RtTg). Through a comprehensive investigation of the neuronal activities of these subpopulations and their contributions to LID, we revealed that these two projection-specific subsets played distinct roles in the development of LID behaviors and were intricately integrated within separate neural pathways. We verified the direct impact of STN activity on LID, which has substantial implications for the development of precise modulation strategies in the treatment of LID.

## RESULTS

### Divergent efferent pathways of STN neurons

**Fig. 1.**
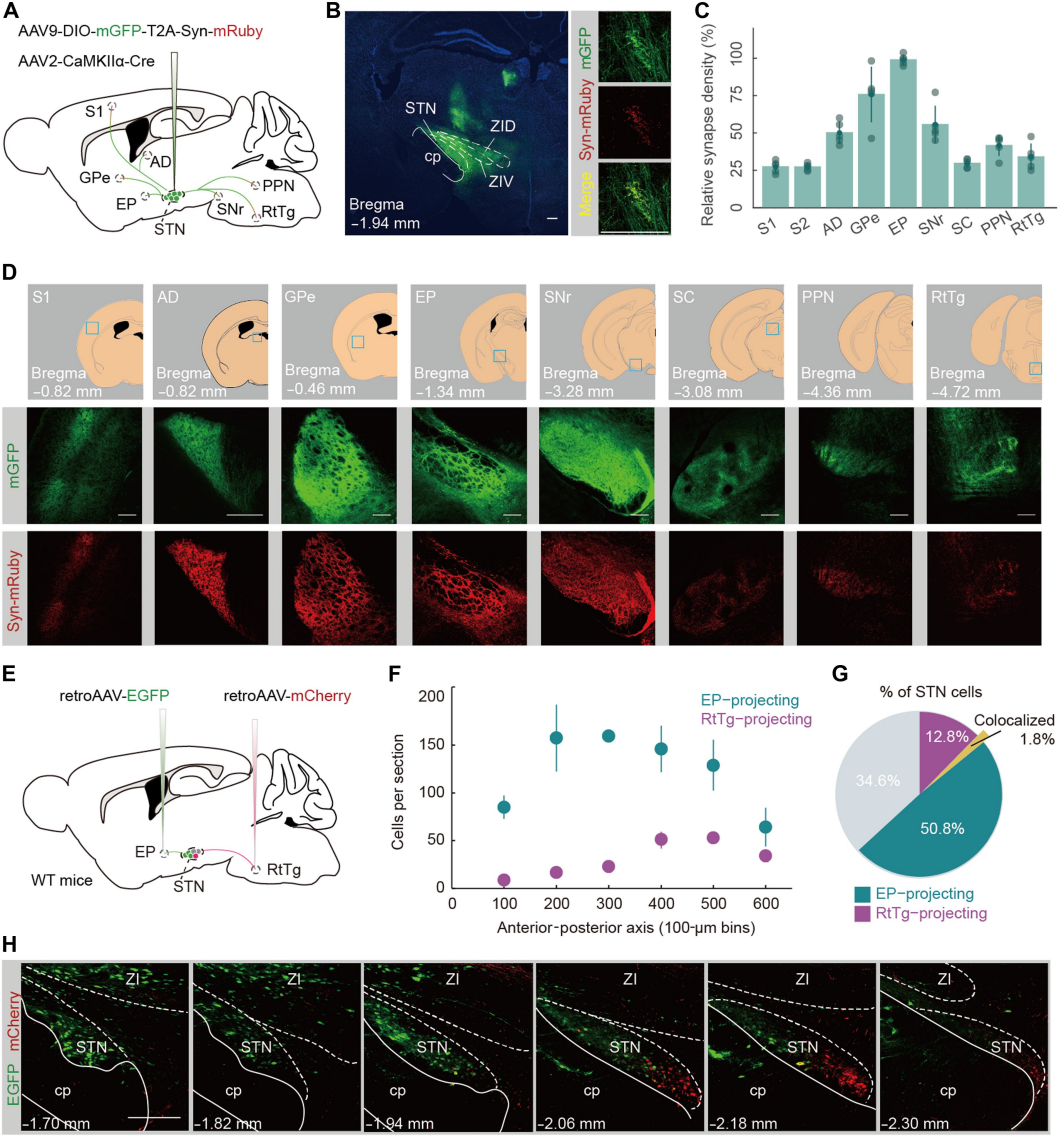
STN^EP^ and STN^RtTg^ neurons are anatomically distinct subpopulations. (**A**) Viral strategy to map the whole brain output of STN neurons. adeno-associated virus 2 (AAV2)–CaMKIIα–Cre and AAV9-DIO-mGFP-T2A-Syn-mRuby were injected into the STN of wild-type (WT) C57BL/6 J mice. (**B**) Example coronal section showing viral expression in the STN and axonal projections toward downstream regions. Insets show mGFP (soma, axonal fibers, green), Syn-mRuby (presynaptic sites, red), and merged signals. Scale bars, 200 μm. (**C**) Relative synapse density of nine primary targets of STN neurons. *n* = 5 mice. (**D**) Representative coronal images of axonal projections (mGFP) and synaptic terminals (Syn-mRuby) in indicated brain regions at corresponding bregma levels. Scale bars, 200 μm. (**E**) Viral strategy to simultaneously label EP-projecting STN neurons (STN^EP^) with EGFP and RtTg-projecting STN neurons (STN^RtTg^) with mCherry. (**F**) Cell counts of STN^EP^ and STN^RtTg^ neurons per representative sections of the STN. *n* = 5 mice. (**G**) Percentage distribution of different subpopulations of STN cells. *n* = 5 mice. (**H**) Representative images of the topological distribution of STN^EP^ and STN^RtTg^ neurons along the anterior-posterior axis of the STN. Scale bars, 200 μm. All data are shown as means ± SEM.

To map the whole-brain outputs of STN neurons, we injected an adeno-associated virus (AAV) with Cre-dependent expression of a green fluorescent protein [monomeric green fluorescent protein (mGFP)] and synaptophysin fused with an enhanced red fluorescent protein (mRuby), along with AAV-CaMKIIα-Cre, into STN of wild-type C57BL/6 J mice (Fig. 1A). Calcium- and calmodulin-dependent protein kinase IIα (CaMKIIα) is widely expressed in the STN and shows extensive colocalization with Vglut2, a selective marker for glutamatergic neurons (fig. S1G). With the mRuby expression restricted to the presynaptic terminals, this approach allowed us to distinguish synaptic targets from the axon of passage. Consistent with previous findings (*22*–*24*), we observed strong labeling of synaptic terminals in several regions, including the EP (homologous to the GPi in primates), GPe, SNr, the anterodorsal thalamic nucleus (AD), the pedunculotegmental nucleus (PPN), and RtTg (Fig. 1, A to D). Notably, considering the variable synaptic density among animals, it is plausible that the fluorescent-labeled neurons in each experiment represent distinct subsets of STN neurons that are spatially separated with specific projection patterns. To identify these subsets of STN neurons with co-collateralization, we performed a pairwise correlation and hierarchical clustering analysis, focusing on the top nine targets of STN (table S3) (*25*). Hierarchical clustering of projection intensity correlations revealed that the nine downstream targets segregated into distinct clusters, with projections to EP and RtTg clustering separately from other targets (fig. S1H). The EP, which is the output of the basal ganglia, is known to exhibit reduced activity during hyperkinesia and serves as a classic DBS target for dystonia and dyskinesia (*26*–*28*). The RtTg is a brainstem structure at the mesencephalic-pontine junction that serves as a major precerebellar nucleus and is involved in sensorimotor integration, and is shown to be substantially activated during the initiation of the startle reflex (*29*, *30*). Given these distinct functional characteristics and hierarchical tendencies, we hypothesize that separate subsets of STN neurons project either to the EP or the RtTg.

To test this hypothesis, we used a retrograde virus tracing technique to label STN^EP^ and STN^RtTg^ neurons with enhanced green fluorescent protein (EGFP) and mCherry fluorescent proteins, respectively (Fig. 1E). The proportion of STN neurons exhibiting collateral projections to both EP and RtTg was relatively low (1.8%), which could be due to the limited labeling efficiency of the retro-AAV. Nevertheless, we observed distinct spatial distributions of the cell bodies of STN^EP^ and STN^RtTg^ neurons within the STN. STN^EP^ neurons were primarily located in the dorsal-anterior regions, whereas STN^RtTg^ neurons were predominantly situated in the ventral-posterior regions (Fig. 1, F to H). These results indicate that STN^EP^ and STN^RtTg^ neurons are two anatomically distinct subpopulations.

### STN^EP^ neurons are selectively activated during LID

**Fig. 2.**
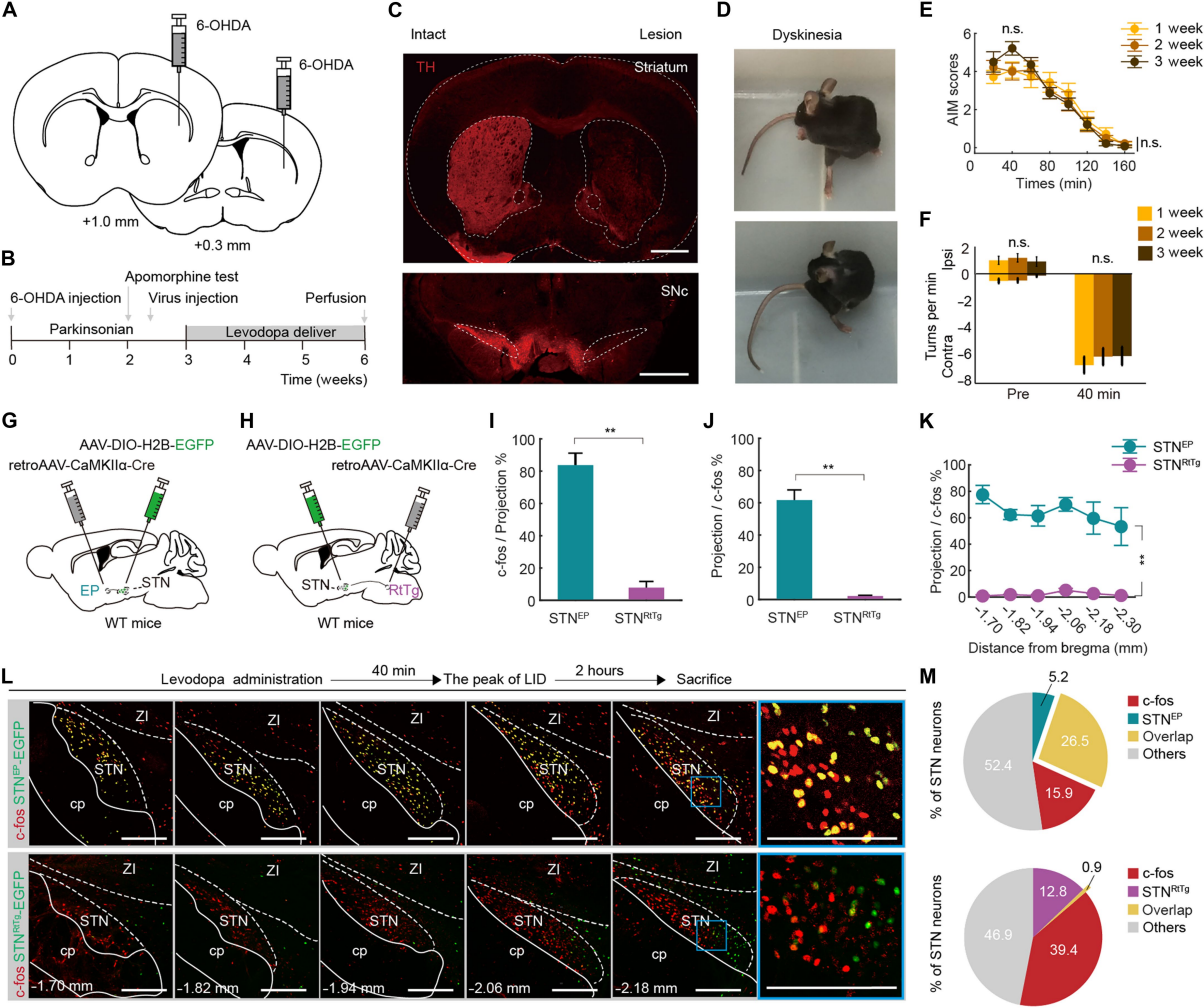
Comparison of the activities of STN^EP^ and STN^RtTg^ neurons during LID using c-fos staining. (**A**) Schematic of 6-OHDA injection sites. (**B**) Timeline of LID mouse model development. (**C**) Representative images of striatal tissue (top) and substantia nigra tissue (bottom) stained with anti-TH. Scale bar, 1 mm. (**D**) Representative images showing LID in LID mice. (**E**) Average AIM scores after levodopa injection over 1 to 3 weeks of levodopa exposure [*n* = 8 mice. n.s., nonsignificant; two-way analysis of variance (ANOVA)]. (**F**) Comparison of rotations and torsions over 1 to 3 weeks of levodopa exposure for the preinjection and LID peak phase (40 min after levodopa injection). *n* = 8 mice. One-way ANOVA. (**G** and **H**) Schematic of the approach to label STN^EP^ (G) and STN^RtTg^ (H) neurons in LID mice. (**I**) Percentage of c-fos^+^ neurons in the STN^EP^ and STN^RtTg^ subpopulation. (*n* = 5 mice for each group. **P < 0.01; Welch’s *t* test) (**J**) Percentage of projection-specific neurons in the total c-fos^+^ neurons (*n* = 5 mice for each group. **P < 0.01; Welch’s *t* test). (**K**) Distribution of the percentage of projection-specific neurons on the anterior-posterior axis of the STN among the corresponding c-fos^+^ neurons. (*n* = 5 mice for each group. **P < 0.01; two-way ANOVA). (**L**) Histological images showing the overlap of c-fos^+^ neurons and STN^EP^ (or STN^RtTg^) neurons across the anterior-posterior axis of STN. The right panels show higher-magnification views of the boxed regions, with merged fluorescence signals indicating cellular colocalization. Scale bar, 200 μm. cp, cerebral peduncle. (**M**) Proportion of c-fos^+^, projection-specific, and overlapping neurons among all STN neurons (top; *n* = 5226 neurons, *n* = 2 mice; bottom; *n* = 3484 neurons, *n* = 3 mice). All data are shown as means ± SEM. Statistics detailed in table S2.

Given the distinct topological organization of STN^EP^ and STN^RtTg^ neurons, we wondered whether their activities differ during LID. To establish a LID mouse model, we performed unilaterally injections of 6-hydroxydopamine (6-OHDA) into two sites within the dorsal-lateral striatum (*31*) (Fig. 2A). After 2 weeks, mice exhibiting positive results in the apomorphine test proceeded to receive systemic levodopa administration for 3 to 6 weeks postsurgery (Fig. 2B). Immunostaining of slices from LID mice with anti–tyrosine hydroxylase (TH) confirmed a near-complete depletion of nigrostriatal DA neurons and fibers on the lesioned side, with intact TH expression on the contralateral side (Fig. 2C). Dyskinesia was quantified using a validated rating scale for AIMs (*32*). Parkinsonian mice displayed dyskinesia, which was characterized by peak intensity occurring 20 to 60 min after levodopa injection and accompanied by contralateral torsion (Fig. 2, D to F). The induction of LID was reliable throughout the 3 weeks of treatment (Fig. 2, E and F).

We next examined the activation of STN^EP^ and STN^RtTg^ neurons in LID mice using c-fos immunostaining. To selectively label STN^EP^ or STN^RtTg^ neurons, we injected retroAAV-CaMKIIα-Cre into EP or RtTg on the lesioned side, along with AAV-DIO-H2B-EGFP into the ipsilateral STN, at the time point between the apomorphine test and the beginning of levodopa delivery (Fig. 2, B, G, and H). Mice were euthanized 2 hours after levodopa injection, and c-fos–positive neurons were considered as those activated at the peak of LID. We found that ~40% of total STN neurons were c-fos positive (Fig. 2M), with a predominant localization in the dorsal-lateral part of STN (Fig. 2L). Notably, the specificity of c-fos expression depended on the projection subpopulations rather than spatial distribution (Fig. 2K). The majority of STN^EP^ neurons (83% ± 7.4%) exhibited c-fos immunoreactivity, and a marked proportion of c-fos–positive neurons (63% ± 5.3%) corresponded to STN^EP^ neurons (Fig. 2, I and J). In contrast, there was minimal overlap between c-fos–positive neurons and STN^RtTg^ neurons (Fig. 2, I to M). Thus, the results demonstrate that STN^EP^ neurons, but not STN^RtTg^ neurons, are selectively activated during LID state.

### Levodopa enhances the ensemble activity of the STN

Before delving into the subpopulations within the STN, it is essential to understand the ensemble activity of STN. STN activity can both modulate locomotion and be modulated by locomotion (*8*). While local stimulation in the STN often elicits milder dyskinesia compared with LID, it is noteworthy that subtle dyskinetic behavior has been observed even at low doses of levodopa in rodent models (*4*). We thus first examined whether the LID state could be differentiated from the “on” state of medicine in the rodent models.

We distinguished different kinetic states in an additional cohort of mice by administering varying doses of levodopa over three consecutive days. The behavior of the mice was recorded using two cameras positioned orthogonally (fig. S2A). Each state exhibited distinct kinematic signatures in terms of dyskinesia and locomotion (fig. S2, B and C). The dyskinesia state was triggered by the administration of high-dose levodopa, leading to involuntary hyperkinetic and dystonic movements characterized by rapidly rising and progressively falling AIMs, with an improvement in locomotion following the apparent phase of LID (fig. S2B, left). The subdyskinesia state was induced by administering a low-dose levodopa to mimic the clinical “on” state. By carefully adjusting the dosage to elicit only subtle dyskinetic behavior, subdyskinetic dose of levodopa resulted in contralateral rotation and velocity increase that were positively correlated [correlation coefficient (*r*) = 0.86, *P* < 0.000; fig. S2D], possibly reflecting spontaneous motor performance (fig. S2B, middle). Parkinsonian mice injected with equal amounts of saline exhibited transient ipsilateral rotations and a decrease in velocity, indicating habituation to the environment (fig. S2B, right).

**Fig. 3.**
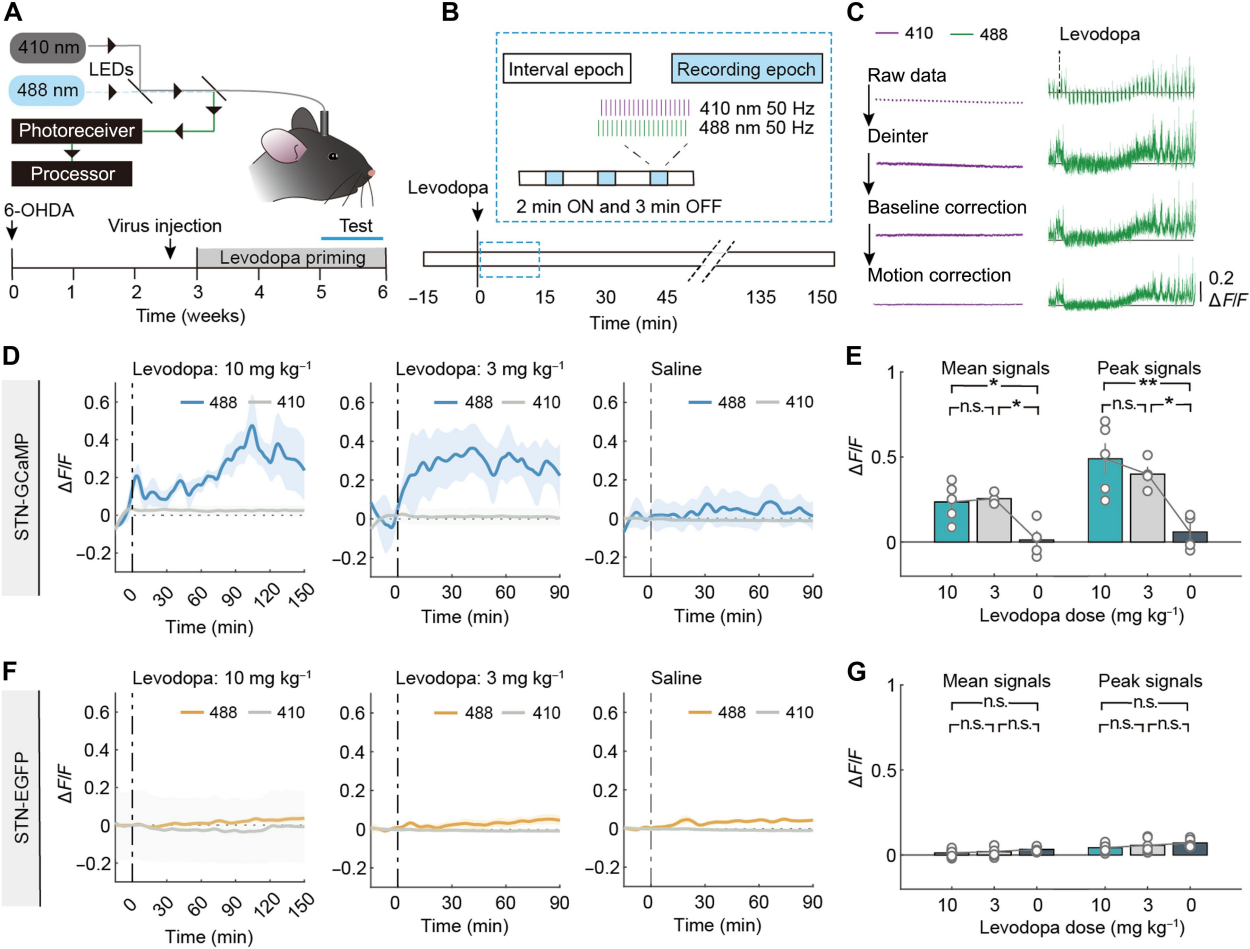
L-DOPA nonmonotonically increases the ensemble activity of the STN. (**A**) Schematic of fiber photometry setups and recording timeline in levodopa-primed mice. LEDs, light-emitting diodes. (**B**) Fiber photometry recording procedure. Alternating 410- and 488-nm lasers of 2-min duration were used every 5 min during a session. (**C**) Representative example for preprocessing analysis of GCaMP signal in an interval recording design. The Δ*F*/*F* signal before levodopa injection was corrected to zero. (**D** and **F**) Long-term changes in GCaMP (D) and EGFP (F) signal of STN neurons in response to levodopa administration at different doses. (**E**) The corresponding mean or peak of the STN global calcium signal (*n* = 5 Vglut2-Cre mice for 10 mg/kg, *n* = 4 Vglut2-Cre mice for 3 and 0 mg/kg; *P < 0.05 and **P < 0.01; one-way ANOVA). (**G**) The corresponding mean or peak of the EGFP signal (*n* = 13 Vglut2-Cre mice for 10 mg/kg, *n* = 8 Vglut2-Cre mice for 3 mg/kg, *n* = 9 Vglut2-Cre mice for 0 mg/kg. One-way ANOVA). All data are shown as means ± SEM. Statistics detailed in table S2.

Given the distinct signatures of kinetic state, we next determined whether the ensemble activity of STN displayed dynamic changes to dyskinetic and locomotor factors. We injected an AAV containing the Cre-dependent Ca^2+^ indicator GCaMP7s into the STN of Vglut2-ires-Cre mice and used fiber photometry to measure the ensemble calcium activity during each state (Fig. 3A). To minimize potential confounding effects of long-term recording on calcium signal levels, we adopted an interval recording strategy, with 2-min recording epochs interleaved with 3-min intervals (Fig. 3, B and C). For the analysis of calcium activity, AIM rating, and motor kinematics, we focused on the middle 1-min segment of each recording epoch. Before each session for a specific kinetic state, there was a habituation period of at least 15 min to stabilize motion. Following the habituation period, either a high or low dose of levodopa or saline was administered. The calcium signal recorded before the administration served as the baseline for subsequent analysis.

Our results revealed an overall increase in fluorescence intensity during both the LID state and the subdyskinesia state compared with the parkinsonian state (Fig. 3, D and E). Increasing the dose of levodopa did not lead to significant differences in calcium activity between the LID and subdyskinesia state, as indicated by both the mean and peak signal (Fig. 3E). Notably, during the LID state, the fluorescence signal initially rose, then decreased, and subsequently increased again, exhibiting a U-shaped profile that persisted for ~90 min throughout the apparent dyskinesia phase (Fig. 3D, left). In contrast, the STN ensemble activity during the subdyskinesia state maintained a relatively stable pattern (Fig. 3D, middle). To assess potential nonspecific effects of motion and fluorescence noise, we included a control group injected with viruses lacking the Ca^2+^ indicator component. In the control mice, we did not observe any significant dynamic changes throughout the entire experimental period (Fig. 3, F and G). These findings suggest that spatially or temporally specific inhibition may play a role in the nonmonotonical modulation of overall STN activity in response to high-dose levodopa.

### Excitation of ensemble STN rescues dyskinetic behavior

**Fig. 4.**
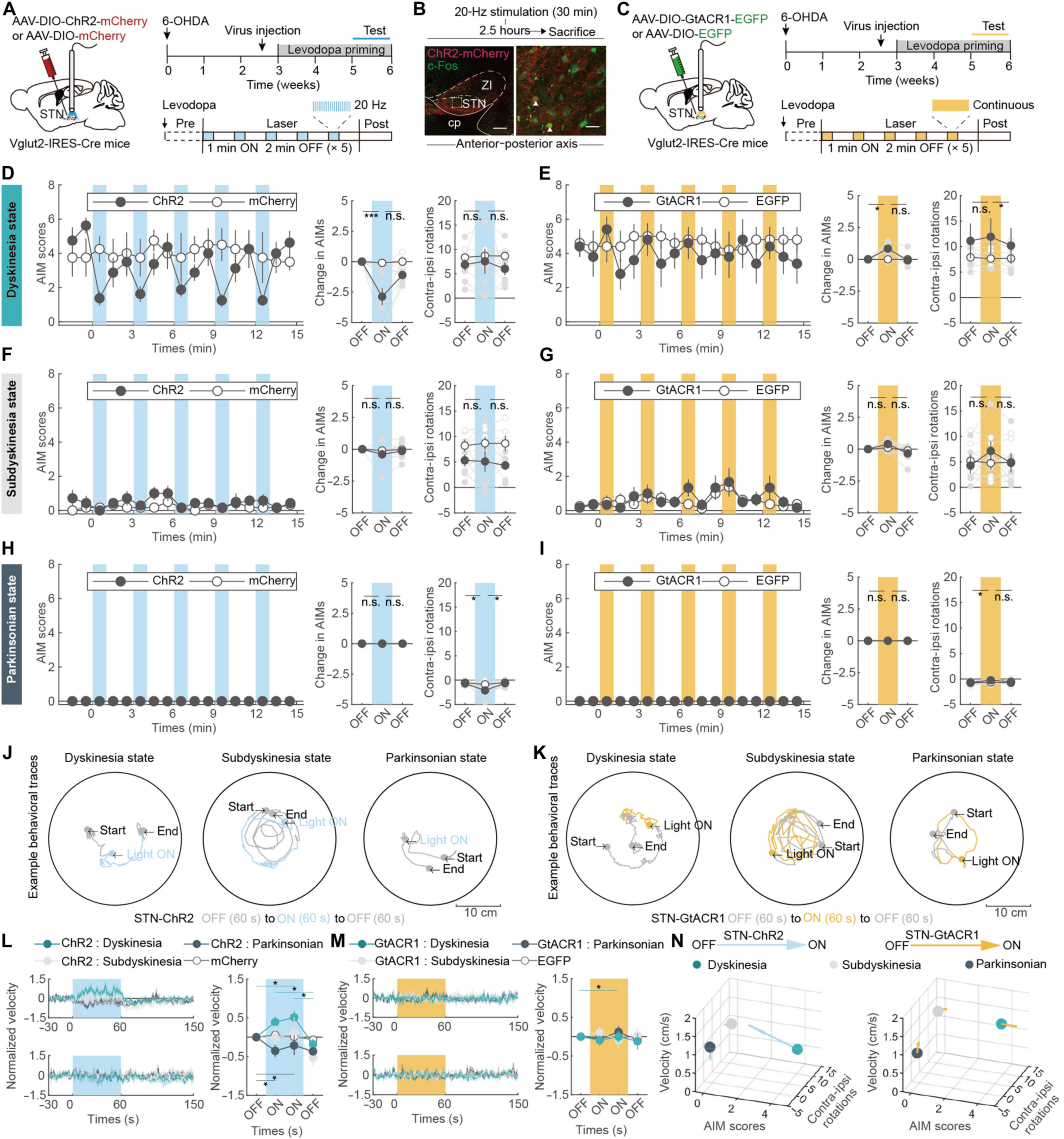
Optogenetic manipulation of STN excitatory neurons. (**A** and **C**) Schematic showing viral strategy, optic fiber implantation, and timeline for optogenetic activation (A) or inactivation (C) protocol in levodopa-primed mice. (**B**) Representative histology showing virus injection and optic fiber placement. Scale bars, 200 μm. (**D** and **E**) Effects of activation [(D); *n* = 8 ChR2, *n* = 4 mCherry. ***P < 0.001] and inactivation [(E); *n* = 5 GtACR1, *n* = 5 EGFP. *P < 0.05 two-tailed paired *t* test] of STN excitatory neurons on AIM scores, changes in AIM score, and rotation bias during the LID state. (**F** and **G**) Effects of activation [(F); *n* = 7 ChR2, *n* = 6 mCherry. n.s.] and inactivation [(G); *n* = 6 GtACR1, *n* = 8 EGFP. n.s.; two-tailed paired *t* test] in the subdyskinetic state. (**H** and **I**) Effects of activation [(H); *n* = 7 ChR2, *n* = 6 mCherry. *P < 0.05] and inactivation [(I); *n* = 7 GtACR1, *n* = 6 EGFP. *P < 0.05; two-tailed paired *t* test] in the parkinsonian state. (**J** and **K**) Representative locomotor trajectories during light-on and light-off epochs following STN activation (J) or inactivation (K) across motor states. (**L** and **M**) Effects of activation [(L); *n* = 8 ChR2, *n* = 4 mCherry. *P < 0.05, two-tailed paired *t* test] and inactivation [(M); *n* = 5 GtACR1, *n* = 5 EGFP. *P < 0.05; Wilcoxon test] on normalized velocity across states. (**N**) Demonstration of the overall effect of optogenetic manipulation of STN on different kinetic states. All data are shown as means ± SEM. Statistics detailed in table S2.

Since the activity of STN ensemble exhibited a U-shaped curve during dyskinesia, implying a possible coexistence of excitation and inhibition, we next determined the role of the STN ensemble through direct manipulation. We used optogenetic modulation of STN activity using a similar experimental setup (fig. S2A). Given that most of the STN neurons are excitatory, we injected AAV-DIO-ChR2-mCherry or AAV-DIO-GtACR1-EFGP (using AAV-DIO-mCherry or AAV-DIO-EGFP as control) into the STN of Vglut2-Cre mice (Fig. 4, A and C, left). The viral efficacy was validated through in vivo electrophysiology (fig. S3). The optogenetic experiments were performed 5 to 6 weeks after systemic modeling treatment (Fig. 4, A and C, top). During different kinetic states, the mice were monitored for AIMs and locomotor kinematics in response to unilateral light activation. Each session for a specific kinetic state consisted of a 3-min habituation period, followed by the administration of either high- or low-dose levodopa or saline. The optogenetic effect was evaluated during the 20- to 40-min period when the LID state was apparent. To avoid the ceiling effect, we delivered an adjusted dose of levodopa (6 to 10 mg kg^−1^) during the LID state. Optical stimulation was performed in blocks of laser-on (1-min duration, 10-ms square pulses at 20 Hz, 488-nm blue light at 6 mW) and laser-off (2 min) epochs, which were repeated five times. Data analysis was performed every minute (Fig. 4, A and C). The stimulation paradigm for optical excitation was determined by testing the effectiveness of various stimulation frequencies (ranging from 5 to 120 Hz) (fig. S3, C to F). A frequency of 20 Hz, which effectively drove spiking activity of STN neurons in vivo, was selected for use throughout this study. Postmortem staining for c-fos was performed to confirm the optical excitation in each mouse (Fig. 4B).

In the dyskinesia state, optogenetic excitation of STN Vglut^2+^ neurons led to a significant reduction in AIM scores compared with the prelaser epoch, as well as a decrease in axial and limb subscores (Fig. 4D and fig. S4). This intervention effectively alleviated axial torsion without affecting rotation bias. Notably, when comparing AIM scores between the laser-on and postlaser epochs, no rapid rebound effect was observed following the cessation of stimuli (Fig. 4D; fig. S4A, top; and movie S1). This improvement in dyskinesia was accompanied by a notable increase in velocity during laser activation (Fig. 4, J and L, and fig. S4A, bottom). No significant effects of light stimulation were observed in the control group (Fig. 4, D and L). Unexpectedly, during the other two states, excitation of STN Vglut^2+^ neurons did not elicit noticeable changes, except for a modest reduction in rotation and velocity during laser-on compared with the prelaser and postlaser epochs in the parkinsonian state (Fig. 4, F, H, and L, and figs. S5A and S6A). Conversely, inhibition of STN Vglut^2+^ neurons induced a modest but significant increase in AIMs during the LID state (Fig. 4E; fig. S4B, top; and movie S2). This effect appeared to be levodopa dose dependent, as only minor changes in AIMs and locomotor kinematics were observed in the subdyskinesia or parkinsonian state, similar to the effect observed in the excitation experiment (Fig. 4, G, I, and M, and figs. S5B and S7A).

Together, these results demonstrate that the dyskinesia state, specifically the involuntary movements, can temporally transition to a subdyskinesia pattern when the STN ensemble is activated, and the dyskinesia symptoms may be exacerbated by STN inhibition (Fig. 4N). The results suggest a conditional motor suppression effect of the STN ensemble, which may facilitate voluntary movement in the presence of redundant levodopa.

### STN^EP^ and STN^RtTg^ neurons display distinct dynamic patterns of activity during LID

**Fig. 5.**
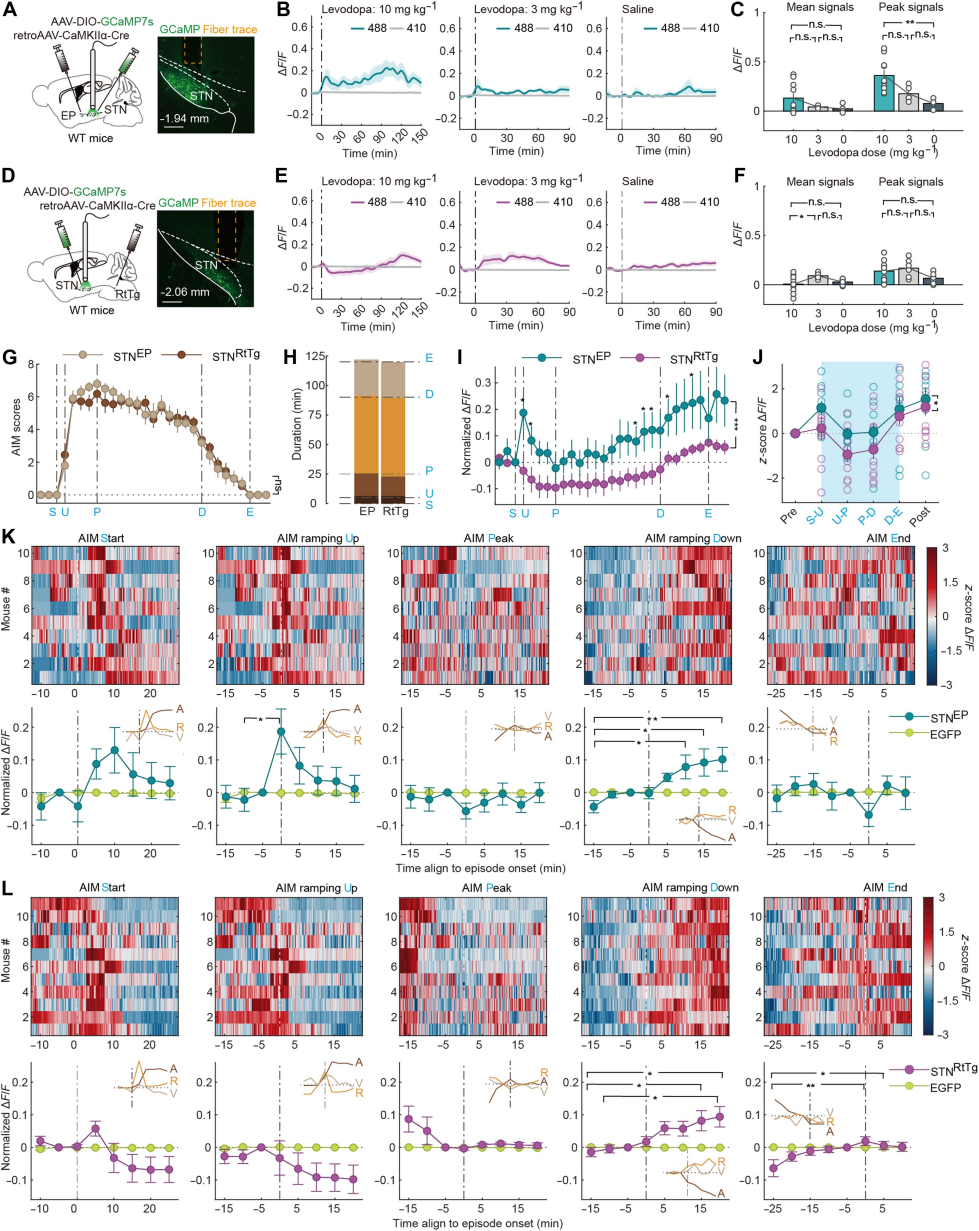
STN^EP^ and STN^RtTg^ neurons show different patterns of calcium response measured by fiber photometry during a LID attack. (**A** and **D**) Schematic of viral targeting and fiber photometry recording of STN ^EP^ (A) and STN ^RtTg^ (D) neurons. Representative images show GCaMP expression and fiber placement. Scale bar, 200 μm. (**B** and **E**) Long-term GCaMP signals in STN ^EP^ [(B); *n* = 10, 7, 6 for 10, 3, and 0 mg/kg L-DOPA] and STN ^RtTg^ neurons [(E); *n* = 11, 8, 9 for 10, 3, and 0 mg/kg L-DOPA] following levodopa administration at different doses. (**C** and **F**) Quantification of mean/peak calcium signals in STN ^EP^ [(C); **P < 0.01] and STN ^RtTg^ neurons [(F); *P < 0.05; one-way ANOVA]. (**G**) Averaged AIM scores curves during high-dose levodopa sessions (*n* = 10 STN ^EP^, *n* = 11 STN ^RtTg^. ns; two-way ANOVA). The onset of dyskinesia episodes: start (S), ramp-up (U), peak (P), ramp-down (D), and end (E). (**H**) Distribution of dyskinesia episodes across groups. (**I** and **J**) Calcium dynamics aligned to dyskinesia episodes, shown as normalized Δ*F*/*F* (I) and *z*-scored signals (J) (*n* = 10 STN ^EP^, *n* = 11 STN ^RtTg^. *P < 0.05, ***P < 0.001; two-way ANOVA). (**K** and **L**) Heatmaps of *z*-scored Δ*F*/*F* aligned to dyskinesia episodes in STN ^EP^ [(K); *n* = 10] and STN ^RtTg^ neurons [(L); *n* = 11], with corresponding averaged traces below (*P < 0.05, **P < 0.01; one-way ANOVA). Insets show aligned behavioral parameters: AIM scores (A), rotation bias (R), and velocity (V). All data are presented as means ± SEM. Statistics detailed in table S2.

Given their distinct projection patterns and c-fos response to LID, STN^EP^ and STN^RtTg^ neurons likely convey separate information during kinetic state. We next measured the activity of STN^EP^ or STN^RtTg^ neurons during each state using fiber photometry. We injected AAV-DIO-GCaMP7s into STN on the 6-OHDA–lesioned side, and retroAAV-CaMKII-Cre into the ipsilateral EP or RtTg (Fig. 5, A and D). We observed distinct patterns in the activity of STN^EP^ and STN^RtTg^ neurons across different states. STN^EP^ neurons exhibited an overall increase in activity compared with the baseline, characterized by a U-shaped curve, with a significantly higher peak signal compared with the parkinsonian state. This suggests a specific response associated with involuntary movement, as no significant changes in activity were observed during the subdyskinesia or parkinsonian state (Fig. 5, B and C). In contrast, STN^RtTg^ neurons initially exhibited persistent inhibition relative to the baseline, followed by a gradual increase in activity (Fig. 5E). Notably, during the subdyskinesia state, STN^RtTg^ neurons showed overall excitation, with the mean signal significantly higher than that of the LID state (Fig. 5, E and F). This excitation may be linked to an increase in voluntary locomotion, as indicated by the concurrent enhancement in locomotor kinematics (Fig. 5L).

To characterize the temporal dynamics of STN^EP^ and STN^RtTg^ ensemble activity during dyskinesia, we aligned the calcium signal with the onset of dyskinetic episodes. On the basis of the corresponding AIM score curve, we identified five onset points for the dyskinesia episodes in each mouse (Fig. 5G): (i) AIM Start onset, which corresponds to the recording epoch preceding the administration; (ii) AIM Ramping up onset, which corresponds to the recording epoch with the maximum slope during the ascending phase of the AIM curve; (iii) AIM Peak onset, which corresponds to the recording epoch at the peak of the AIM curve; (iv) AIM Ramping down onset, which corresponds to the recording epoch with the maximum slope during the descending phase of AIM curve; (v) AIM End onset, which corresponds to the recording epoch when the AIM curve returned to a score of zero. To account for variations in the total duration and proportion of dyskinesia episodes among mice, we corrected the episode onsets on the basis of the average distribution. For the two STN subpopulations, the overall AIM score curves did not show any observable or statistically significant differences after the correction, and the distributions of the time course were similar (Fig. 5, G and H). However, despite the similarity in the AIM score curves, the calcium signal curves differed significantly between the STN subpopulations, particularly during the AIM Ramping up and AIM Ramping down episodes (Fig. 5, I and J). Upon aligning the calcium signal to the episode onsets, we observed that the STN^EP^ ensemble exhibited brief activation during the AIM Ramping up and Ramping down episodes, and the activation was accompanied by rapid behavior switching as indicated by both AIM scores and rotation (Fig. 5K). On the other hand, the STN^RtTg^ ensemble displayed a transient and nonsignificant activation following the administration, with a more pronounced excitation occurring around the AIM Ramping down to AIM End episode, possibly reflecting the rotation and velocity changes associated with increased voluntary movement (Fig. 5L).

To elucidate the relationship between ensemble activity of the STN^EP^ and STN^RtTg^ subpopulations and dyskinesia episodes, we conducted correlation analyses between the calcium signal and various behavioral variables, including AIM scores, rotation times, and velocity, as well as time (figs. S8 and S9). For both the STN^EP^ and STN^RtTg^ subpopulations, we found that their activity exhibited a negative correlation with AIM scores and a positive correlation with locomotor kinematics during the dyskinesia period (figs. S8, A and C, and S9, A and C). Notably, the correlation coefficients consistently exceeded the partial correlation coefficients, indicating that the ensemble activity was generally influenced by more than one variable (figs. S8, B and D, and S9, B and D). For the STN^EP^ ensemble activity in each episode, when we calculated partial correlation coefficients between the normalized Δ*F*/*F* and behavioral variables, controlling for the remaining variables, we found significant correlations for the three episodes occurring after the onset of dyskinesia (fig. S8, H, J, and L). On the other hand, although the STN^RtTg^ ensemble activity displayed stronger correlations with the majority of behavioral factors during the dyskinesia period, no significant correlation with AIM scores was observed after controlling for other factors in each phase of dyskinesia (fig. S9, F, H, J, and L). This suggests that the activity of the STN^RtTg^ ensemble is influenced by various behavioral variables beyond the AIM scores.

Together, these findings reveal that the activity of STN undergoes dynamic modulation throughout the dyskinesia episodes. The STN^EP^ subpopulation exhibits dynamic changes specifically associated with the switching phase of involuntary movement, whereas the STN^RtTg^ subpopulation displays a broader involvement in the modulation of dyskinesia, potentially reflecting its role in the motivation or maintenance of voluntary movement.

### STN^EP^ and STN^RtTg^ ensembles oppositely regulate LID

**Fig. 6.**
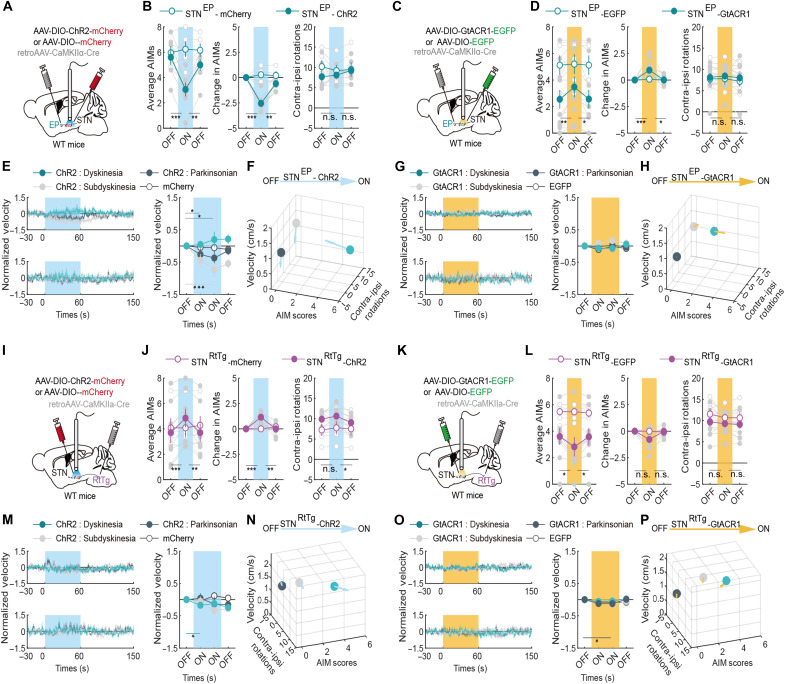
Optogenetic manipulation of STN^EP^ and STN^RtTg^ neurons differentially regulates LID. (**A** and **C**) Viral strategy and fiber implantation for optogenetic activation (A) or inactivation (C) of STN^EP^ neurons. (**B** and **D**) Effects of activating [(B); *n* = 7 ChR2, *n* = 5 mCherry. **P < 0.01, ***P < 0.001] and inactivating [(D); *n* = 11 GtACR1, *n* = 6 EGFP; *P < 0.05, **P < 0.01, ***P < 0.001; two-tailed paired *t* test or Wilcoxon test] STN^EP^ neurons on AIM scores, changes in AIM score, and rotation bias during the LID state. (**E** and **G**) Effects of activating [(E); *n* = 7 ChR2, *n* = 5 mCherry. *P < 0.05, ***P < 0.001] and inactivating [(G); *n* = 11, 12, 14 GtACR1, *n* = 6 EGFP; Wilcoxon test] STN^EP^ neurons on normalized velocity across dyskinetic, subdyskinetic, and parkinsonian states. (**F** and **H**) Overall effect of STN^EP^ manipulation across kinetic states. (**I** and **K**) Viral strategy and fiber implantation for optogenetic activation (I) or inactivation (K) of STN^RtTg^. (**J** and **L**) Effects of activating [(J); *n* = 8 ChR2, *n* = 5 mCherry. ns, *P < 0.05, **P < 0.01, ***P < 0.001] and inactivating [(L); *n* = 9 GtACR1, *n* = 6 EGFP. *P < 0.05; two-tailed paired *t* test or Wilcoxon test] STN^RtTg^ neurons on AIM scores, changes in AIM score, and rotation bias during the LID state. (**M** and **O**) Effects of activating [(M); *n* = 8 ChR2, *n* = 5 mCherry. *P < 0.05] and inactivating [(O); *n* = 9, 9, 12 GtACR1; *n* = 6 EGFP. *P < 0.05; Wilcoxon test] STN^RtTg^ neurons on normalized velocity across dyskinetic, subdyskinetic, and parkinsonian states. (**N** and **P**) Overall effect of STN^RtTg^ manipulation across kinetic states. All data are shown as means ± SEM. Statistics detailed in table S2.

Given the different response patterns of STN^EP^ and STN^RtTg^ neurons during LID, we next investigated how the activity of these two subsets modulates kinetic state. Using the retrograde viral strategy, ChR2 or mCherry (as a control) was selectively expressed in STN^EP^ or STN^RtTg^ neurons in levodopa-primed parkinsonian mice (Fig. 6, A and I). Subsequently, we unilaterally photostimulated the ChR2-infected STN neurons while simultaneously monitoring the AIMs and locomotor kinematics of the mice (Fig. 6, A and I). We found that activation of STN^EP^ neurons resulted in a significant decrease in the AIM scores, affecting all three subscores of AIM ratings (Fig. 6B, fig. S4C, and movie S3). During STN^EP^ activation, while the reduction in AIM scores was similar to that observed during STN Vglut^2+^ activation, a rapid rebound effect was observed for both the AIM scores and subscores, almost returning to prelaser levels upon cessation of light stimulation (Fig. 6B). In the dyskinesia state, photoactivation of STN^EP^ alleviated involuntary movement without significantly affecting velocity (Fig. 6, E and F). However, in the subdyskinesia and parkinsonian states, STN^EP^ activation led to a significant decrease in velocity (Fig. 6, E and F, and figs. S5C and S6D). These results indicate that EP-projecting STN neurons are directly involved in modulating movements. Conversely, activation of STN^RtTg^ neurons exacerbated involuntary behaviors in the dyskinesia state, particularly through an increase in orolingual AIM subscores, accompanied by a modest decrease in velocity compared with the prelaser epoch (Fig. 6, J, M, and N; fig. S4E; and movie S4). Optogenetic activation of STN^EP^ or STN^RtTg^ neurons did not significantly affect rotation bias (Fig. 6, B and J). Thus, activation of STN^EP^ and STN^RtTg^ neurons elicits opposing effects on the LID state.

Next, we determined whether the activity of specific STN subsets is necessary for LID. We expressed GtACR1 or EGFP (as a control) in STN^EP^ and STN^RtTg^ neurons (Fig. 6, C and K). In contrast to the effects of optogenetic activation, inhibition of STN^EP^ neurons resulted in a significant increase in AIM scores across all three subscores, displaying a more pronounced effect compared with the inactivation of STN Vglut^2+^ neurons (Fig. 6D, fig. S4D, and movie S5). Conversely, inactivation of STN^RtTg^ neurons led to a modest decrease in the AIM scores (Fig. 6L and movie S6), although statistical significance was not reached for all three subscores (fig. S7F). Moreover, optogenetic inactivation of STN^EP^ or STN^RtTg^ neurons did not alter the rotation bias or velocity (Fig. 6, D, G, L, and O, and fig. S4, D and F). These findings demonstrate the contrasting functions of STN^EP^ and STN^RtTg^ neurons in bidirectional modulation of LID behaviors, with STN^EP^ playing a more direct role in locomotion modulation.

### Roles of STN neurons in the induction of dyskinesia

The above experiments demonstrated that specific subsets of STN neurons can substantially influence the severity of involuntary movements during the LID state. We further investigated whether direct manipulation of STN neurons could induce dyskinesia. To explore this possibility, we first analyzed the effect of photostimulation on AIM scores and locomotor kinematics in both parkinsonian and subdyskinesia states.

We found that activation or inhibition of either STN^EP^ or STN^RtTg^ neurons in the parkinsonian state had no effect on AIM scores (Fig. 6, F, N, H, and P). Both the laser-on and perilaser epochs resulted in zero scores, similar to the effect of activating STN Vglut^2+^ neurons (Fig. 4H and table S2). Activation of STN^EP^ neurons caused a slight increase in ipsilateral rotations (fig. S6C), an effect also observed when STN excitatory neurons were activated (Fig. 4H and fig. S6A). Conversely, inhibiting STN^EP^ neurons led to an increase in contralateral rotations, which was not observed with the inactivation of STN Vglut^2+^ neurons (fig. S7, A and C). Optogenetic manipulation of STN^RtTg^ did not affect rotation (figs. S6E and S7E).

In addition, we examined the impact of photostimulation on locomotion in the parkinsonian state (figs. S6, G to L, and S7, G to L). Locomotion states were classified into three categories on the basis of locomotor kinematics, as previously reported (*33*). Activation of STN Vglut^2+^ neurons significantly decreased ambulation and increased fine movement (fig. S6, I to L), while inhibition of STN Vglut^2+^ neurons slightly increased velocity without altering locomotion states (fig. S7, B and G to L). Consistent with the effects of activating STN Vglut^2+^ neurons, activation of STN^EP^ neurons induced more fine movement and less ambulation (fig. S6, I to L), while inhibiting STN^EP^ neurons led to a decrease in immobility and an increase in fine movement (fig. S7, G to L). In contrast, activation of STN^RtTg^ neurons caused a transient increase in velocity (fig. S6F), while inhibition reduced the percentage of ambulation mainly by decreasing ambulation frequency (fig. S7, F and I). Overall, although manipulation of neither STN^EP^ nor STN^RtTg^ neurons induced dyskinesia in the absence of levodopa, these two subpopulations of STN neurons exhibited opposite effects on locomotion in the parkinsonian state of levodopa-primed mice.

Furthermore, in the subdyskinesia state, the effect of manipulating STN Vglut^2+^ neurons on locomotion appeared to occur after the cessation of light stimulation, with more immobility and less ambulation after activation, and a slight increase in immobility after inhibition (fig. S5M). Inhibition of STN^EP^ neurons had a modest effect on exacerbating involuntary movement without significantly affecting locomotor kinematics (fig. S5, A to F). However, optogenetic activation of STN^EP^ worsened akinesia, as evidenced by a simultaneous reduction in contralateral rotations and velocity during the subdyskinesia state, along with an increase in immobility and a decrease in ambulation (fig. S5, C and N). Activation of STN^RtTg^ neurons resulted in a similar extent of reduction in rotation without a significant effect on velocity (fig. S5E). Although ambulation was disturbed during the laser-on epoch, the larger proportion of ambulation during the session suggests a potential effect of STN^RtTg^ modulation on motivation reinforcement (fig. S5O).

Together, optogenetic manipulation of STN neurons alone is insufficient to trigger dyskinesia in levodopa-primed mice. However, in rodent models, subtle dyskinesia can be observed with a lower dose of levodopa administration, and this effect is further intensified by inhibiting the STN^EP^ neurons. These results suggest that distinct subpopulations of STN neurons use diverse mechanisms to modulate locomotion behavior under different conditions.

### Inputs to STN^EP^ and STN^RtTg^ neurons

**Fig. 7.**
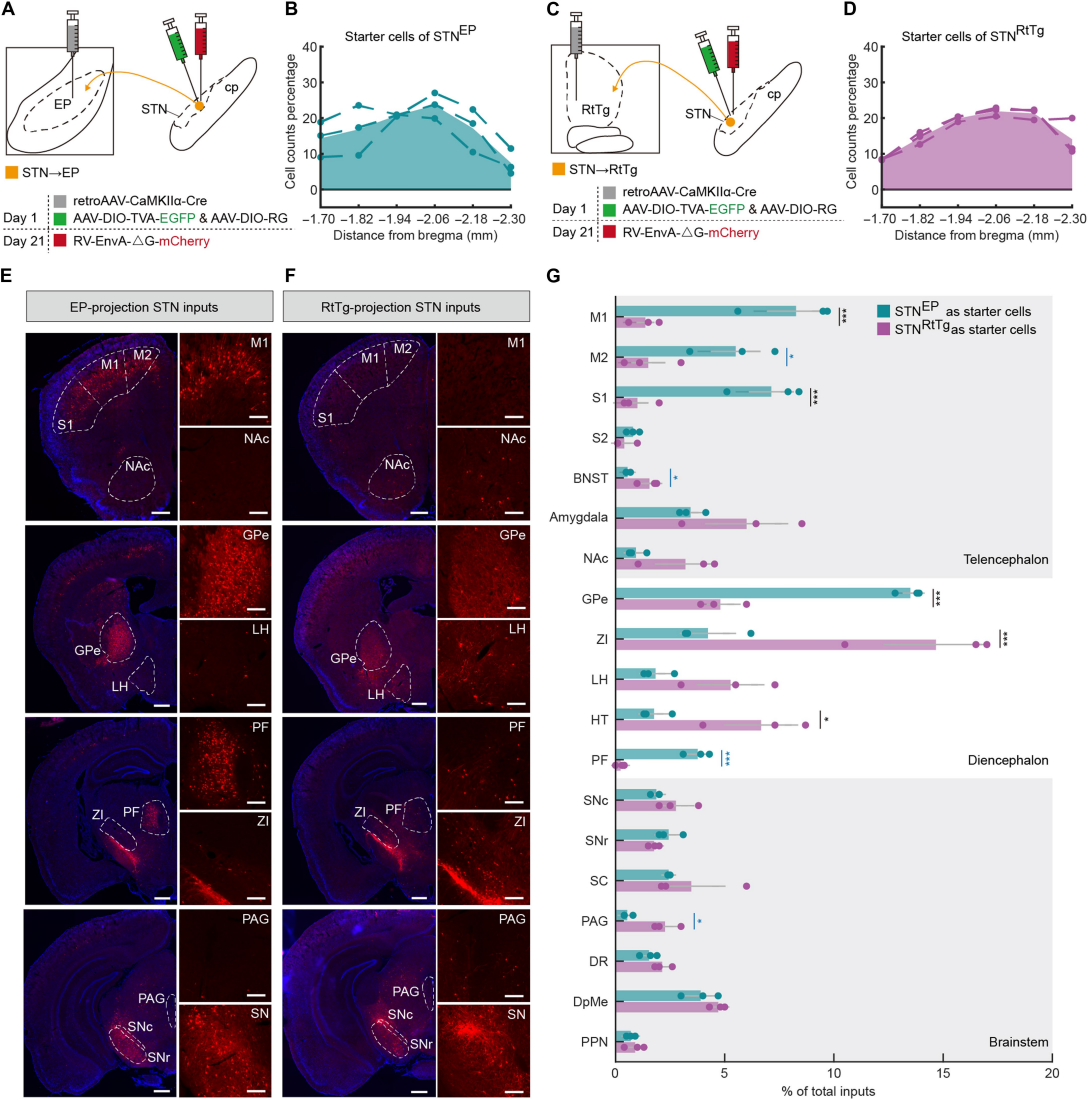
Whole-brain mapping of inputs to STN^EP^ and STN^RtTg^ neurons. (**A**) Experimental protocol for labeling whole-brain inputs to STN^EP^ neurons. (**B**) Starter cell counts of STN^EP^ neurons across the anterior-posterior axis of the STN (*n* = 3 mice). (**C**) Experimental protocol for labeling whole-brain inputs to STN^RtTg^ neurons. (**D**) Starter cell counts of STN^RtTg^ neurons across the anterior-posterior axis of the STN (*n* = 3 mice). (**E** and **F**) Representative images of selected brain areas showing trans-synaptically labeled input neurons of STN^EP^ neurons (E) and STN^RtTg^ neurons (F). Scale bar, 500 μm for left images and 200 μm for right images. (**G**) Whole-brain distribution of inputs to STN^EP^ and STN^RtTg^ neurons. Data are presented as percentage of input cells in each brain area relative to the total number of whole-brain input cells. *n* = 3 mice for each group. *P < 0.05, ***P < 0.001; two-way ANOVA with post hoc Bonferroni correction (black star) and two-tailed *t* test (blue star) for multiple comparisons. M1, primary motor cortex; M2, secondary motor cortex; S1/S2, somatosensory cortex; BNST, bed nucleus of stria terminalis; NAc, accumbens nucleus; LH, lateral hypothalamus; HT, hypothalamus; PF, parafascicular nucleus; SNc, substantia nigra, compact part; SC, superior colliculus; PAG, periaqueductal gray; DR, dorsal raphe; DpMe, deep mesencephalic nucleus; PPN, pedunculopontine nucleus. All data are shown as means ± SEM. Statistics detailed in table S2.

Considering the anatomical and functional distinctions between STN^EP^ and STN^RtTg^ neurons, we reasoned that these two populations may receive inputs from different brain regions. To label the monosynaptic inputs to STN^EP^ or STN^RtTg^ neurons, we injected AAV-CaMKIIα-Cre into EP and RtTg, respectively, followed by an injection of two Cre-dependent helper viruses, AAV-DIO-TVA-EGFP and AAV-DIO-RG, into STN. After 21 days, the EnvA-pseudotyped, glycoprotein-deleted rabies virus (EnvA-RV-ΔG-mCherry) was injected into STN (Fig. 7, A and C). The dual color–labeled neurons were identified as starter cells (fig. S10, A and B). Compared with the starter cells of STN^EP^ neurons, those of STN^RtTg^ neurons were located in a more posterior part of STN (Fig. 7, B and D).

To assess the specificity of the virus tracing, we performed control experiments. No neuron was labeled when we only injected EnvA-RV-G-mCherry into STN or injected AAV-DIO-TVA-EGFP, AAV-DIO-RG, and EnvA-RV-ΔG-mCherry into STN (fig. S10, C to F). In addition, when we injected AAV-DIO-TVA-EGFP and EnvA-RV-ΔG-mCherry into STN with STN^EP^ neurons expressing Cre recombinase, only neurons at the injection site were labeled, and no neuron was labeled outside the injection site (fig. S10, G to I).

To accurately determine the number of input neurons, we excluded neurons located near the injection site. Whole-brain quantification of mCherry-labeled neurons revealed that STN^EP^ neurons received a significantly higher proportion of inputs from sensorimotor areas such as the frontal cortex, GPe, and parafascicular nucleus (PF), known to be involved in motor control. In comparison, STN^RtTg^ neurons received a greater number of inputs from regions including the zona incerta (ZI), hypothalamus, bed nucleus of the stria terminalis (BNST), and periaqueductal gray (PAG), which are implicated in innate behaviors (Fig. 7, E to G). Although both populations received a significant proportion of inputs from GPe, STN^EP^ and STN^RtTg^ neurons showed a preference for inputs from the dorsal GPe and ventral GPe, respectively (Fig. 7, E and F). Moreover, the inputs from the substantia nigra to STN^EP^ and STN^RtTg^ neurons exhibited distinct topological distributions (Fig. 7, E and F).

Together, the results strongly support the notion that STN^EP^ and STN^RtTg^ neurons are essential components of parallel neural pathways. These two subpopulations of neurons likely convey distinct streams of information, potentially related to motion and motivation, to their respective targets, thereby contributing to diverse behavioral functions (*34*, *35*).

## DISCUSSION

It is intriguing that DBS of the STN, originally intended to alleviate akinesia symptoms in PD, also unexpectedly exhibits an antidyskinesia effect. These contradictory outcomes indicate the heterogeneity of STN neurons in regulating LID. Through virtue-mediated tracing, in vivo calcium signal recording, optogenetic manipulation, and behavioral analysis, we have uncovered two distinct subpopulations of STN neurons, which exhibited both topological and functional preferences. The EP-projecting STN neurons, which are distributed in the anterior-dorsal subregion, play a role in direct motion modulation and show strong connectivity with sensorimotor brain regions. In contrast, the RtTg-projecting STN neurons, which are located in the posterior-ventral subregion, receive inputs from limbic brain regions and allow for a broader influence on action guidance.

Previous studies have demonstrated increased activity in STN following dopamine denervation (*36*, *37*). However, the responses of STN neurons to levodopa or apomorphine showed variability among electrophysiological recording studies (*38*–*41*). In our study, we have identified a specific subpopulation of STN neurons, referred to as the ipsi-lesioned STN^EP^ neurons, which are prominently activated during dyskinesia. This finding not only validates previous observations (*42*, *43*) but also provides additional evidence regarding the selective activation within the STN. Furthermore, our results align with previous single-unit recordings, which have reported both increased and decreased changes in neuronal activity compared with the parkinsonian state (*44*, *45*). The heterogeneous activation of STN neurons suggests that the discrepancies observed in previous studies may arise from differences in the subpopulations of neurons recorded, as well as variations in recording methods, state of anesthesia, and levodopa dose administered. While our findings suggest functional divergence of STN subpopulations in dyskinesia, the intrinsic membrane properties underlying this divergence remain unexplored. Subtype-specific electrophysiological recordings will be required to determine whether dopamine denervation differentially alters their intrinsic excitability.

Traditionally, STN neurons have been considered a homogeneous population with parallel inputs distributed along the dorsolateral-to-ventromedial axis of the basal ganglia. Here, we have uncovered a distinct subpopulation within the STN, referred to as STN^EP^, which is within the intra-basal ganglia circuit and occupies most of the STN neurons. These STN^EP^ neurons are widely distributed throughout the entire STN, but exhibit a higher density in the anterior-dorsal region. Rabies virus tracing showed that the monosynaptic inputs to the STN^EP^ subpopulation mainly originated from typical sensorimotor areas, such as the frontal cortex, GPe, and PF. Previous studies have demonstrated that activating the motor cortex and PF caused a hyperkinetic effect. Inhibiting D1 receptors in the motor cortex has been shown to attenuate AIM scores (*46*), and excitation of the PF-STN projection can immediately initiate movement (*47*). In addition, optogenetic activation of GABAergic neurons in the GPe, which inhibit STN neurons, has been shown to induce hyperkinesia in the dopamine-intact mice (*26*). We proposed that these excitatory and inhibitory inputs converge onto the STN^EP^ subpopulation to mediate action control. Our fiber photometry recordings revealed a nonmonotonic modulation of both the overall STN activity and the activity of STN^EP^ during the dyskinesia state, as evidenced by the U-shaped ensemble activity. This dynamic pattern of STN ensemble activity highlights the switching phase of dyskinesia, potentially arising from additional recruitment from these inputs. In comparison, the STN^RtTg^ neurons are relatively restricted to the posterior-ventral region of the STN. The monosynaptic inputs to the STN^RtTg^ neurons originated from extra-basal ganglia nuclei, such as ZI, BNST, and PAG, which are traditionally associated with the limbic system. Notably, ZI and BNST exhibited remarkable increases in immediate early gene immunoreactivity in dyskinetic animals (*48*), and selective inactivation of neurons expressing delta-FosB in the BNST yielded a considerable antidyskinetic effect (*49*). For STN^RtTg^ neurons, we observed a decline in activity during apparent involuntary movements and an increase in activity following the administration of an appropriate dose of dopamine. These findings suggest that the STN^RtTg^ neurons may play a modulatory role rather than directly influencing motor actions, and their primary function may be to accumulate internal perceptual signals to regulate ongoing locomotion. Our result may partly explain the euphoria that can accompany mild dyskinesia and the exacerbation of dyskinesia during stressful emotions in patients with PD. The divergence observed between the two subpopulations of STN during high-dose levodopa administration reflects a gradient of parallel dopamine effects, extending from the motor-related nigrostriatal system to the more valence-related mesocorticolimbic system (*50*). Consequently, the existence of distinct STN subpopulations suggests that the integrated action effects should be considered not only in motor functions but also in nonmotor aspects.

It is important to note that while we designated the two subpopulations as STN^EP^ and STN^RtTg^, it does not imply that STN^EP^ neurons exclusively project to EP and STN^RtTg^ neurons only project to RtTg. A single STN neuron may collaborate with multiple downstream nuclei (*51*–*53*), adding to the complexity of circuit-level interactions. Therefore, our results of soma stimulation represent the collective outcomes of the subpopulation’s influence on all downstream targets, rather than being solely confined to STN-EP or STN-RtTg circuitry. In this context, we found that activating the STN^EP^ subpopulation or inactivating the STN^RtTg^ subpopulation ameliorated LID, whereas inhibiting the STN^EP^ subpopulation or activating the STN^RtTg^ subpopulation exacerbated LID. Notably, stimulation of the STN^EP^ subpopulation caused responses similar to those observed with STN excitatory neurons, whereas stimulation of the STN^RtTg^ subpopulation showed distinct functional characteristics compared with the overall STN neurons. These results may be explained by the unequal proportions of the two subpopulations, as we found that the STN^EP^ subpopulation accounted for approximately two-thirds of the total STN neurons. The ChR2(H134R) variant used here is well suited for low-frequency stimulation, consistent with our experimental paradigm. Faster opsins may allow further dissection of frequency-dependent effects in future studies.

Despite the significance of STN neurons in modulating involuntary movements during dyskinesia state, our optogenetic stimulation of the STN was not sufficient to trigger dyskinesia in the parkinsonian state, albeit with a weaker influence on the akinesia compared with published data (*54*). In contrast, STN lesion or chemogenetic inactivation has been shown to induce hemiballism or dyskinesias in human patients and monkeys (*55*, *56*), although relatively modest effects were observed in subthalamotomy (*57*, *58*). The discrepancy cannot be solely attributed to species difference. Instead, it suggests that the induction of dyskinesia by STN manipulation may be conditional, depending on stimulation frequency or circuit connectivity. It is reported that high-frequency optogenetic stimulation of the STN can elicit hyperkinetic effects (*59*), similar to the effects of low-frequency selective excitation of the PF-STN terminals (*45*). In our study, optogenetic stimulation of the STN^EP^ neurons substantially enhanced involuntary movements with mild dopamine supplementations. Notably, there were no apparent changes in ensemble activity between the subdyskinesia and parkinsonian states. This suggests that the rate model effect is limited in the generation of LID, a notion supported by a lack of association between the STN firing rate and the AIM score (*40*). Instead, modulation of the spatiotemporal profiles or oscillation changes across a broader area may play a role in these processes (*4*, *60*). Our findings indicate that the STN is primarily involved in regulating the maintenance of natural actions rather than initiating LID. Considering that the GPe is the sole GABAergic input to the STN^EP^, our data indicate that the origin of the LID may be initiated by SPNs. This is supported by the observation that inhibition of STN^EP^ was concurrent with apparent LID. In support of this hypothesis, manipulation of dSPNs and iSPNs alone or in combination can elicit dyskinesia without the administration of levodopa in levodopa-primed mice (*3*, *61*, *62*, *63*). Our study provides valuable insights into the complex mechanisms underlying dyskinesia and highlights the multifaceted nature of STN subpopulations. Further research exploring the characteristics of single neurons and the interaction between different neuronal populations will enhance our understanding of LID pathophysiology and open avenues for neuromodulation approaches.

## MATERIALS AND METHODS

### Animals

All experimental procedures were approved by the Animal Care and Use Committee of Fudan University (approval no. JS-414) and performed in accordance with the guidelines of the National Institutes of Health (United States) regarding the care and use of animals. Male adult C57BL/6 J wild-type (Cyagen Biosciences Inc.) and Vglut2-Cre mice (8 to 10 weeks old, 22 to 28 g, one to six animals per cage, the Jackson Laboratory, stock 016963) were maintained on a reversed 12-hour light/dark cycle (lights off at 7:00 a.m.). Behavioral experiments were performed during the daytime. Animals were randomly assigned to different treatment groups.

### Viral vectors and tracers

AAV2/2RetroPlus-CMV_bGI-EGFP-WPRE-pA, AAV2/2RetroPlus-CMV_bGI-mCherry-WPRE-hGHpA, AAV2/9-hEf1a-DIO-EGFP-WPRE-pA, AAV2/9-CaMKII-ChETA–EGFP-ER2-WPRE-pA, AAV2/8-CAG-DIO-GtACR1-p2A-EGFP, AAV2/9-hEF1a-DIO-ChR2(H134R)-mCherry-WPRE-pA, AAV2/9-hSyn-FLEx-mGFP-2A-Synaptophysin-mRuby-WPRE-pA, and AAV2/9-hSyn-DIO-jGCaMP7s-WPRE-pA were purchased from Taitool Bioscience Co. Ltd. (Shanghai, China). AAV2-CaMKIIα-CRE-WPRE-pA, retroAAV-CaMKIIa-Cre-WPRE-pA, AAV2/9-Ef1a-DIO-mCherry-WPRE-pA, AAV2/9-Ef1a-DIO-His-EGFP-2A-TVA-WPRE-pA, AAV2/9-Ef1a-DIO-RVG-WPRE-pA, and RV-EnvA-DG-mCherry were purchased from BrianVTA Co. Ltd. (Wuhan, China).

### Viral constructs and general surgery

To map the whole-brain outputs of STN neurons, 60 nl of AAV2/9-hSyn-FLEx-mGFP-2A-Synaptophysin-mRuby-WPRE-pA mixed with 30 nl of AAV2-CaMKIIα-Cre-WPRE-pA was injected into the STN [coordinates: bregma: anteroposterior (AP) = +1.6 mm; mediolateral (ML) = −1.8 mm; dorsoventral (DV) = −4.6 mm]. To identify the location and overlap of STN^EP^ and STN^RtTg^, 30 nl of AAV2/2RetroPlus-CMV_bGI-EGFP-WPRE-pA and 60 nl of AAV2/2RetroPlus-CMV_bGI-mCherry-WPRE-hGHpA were injected into the EP (coordinates: bregma: AP = +2.0 mm; ML = −1.1 mm; DV = −4.45 mm) and the RtTg (coordinates: bregma: AP = +0.3 mm; ML = −4.17 mm; DV = −5.15 mm), respectively. To examine the activation of STN^EP^ and STN^RtTg^ neurons in LID mice, 50 nl of AAV2/9-Ef1a-DIO-His-EGFP-2a-TVA-WPRE-pA was injected into the STN, 30 and 60 nl of retroAAV-CaMKIIα-Cre-WPRE-pA were injected into the EP and RtTg, respectively.

To observe the calcium signal changes in STN Vglut^2+^ neurons, 50 nl of AAV2/9-hEf1a-DIO-EGFP-WPRE-pA or AAV2/9-hSyn-DIO-jGCaMP7s-WPRE-pA was injected into STN. To measure the activity of STN^EP^ or STN^RtTg^ neurons, 30 and 60 nl of retroAAV-CaMKIIα-Cre-WPRE-pA were injected into the EP and RtTg, respectively; 50 nl of AAV2/9-hEf1a-DIO-EGFP-WPRE-pA or AAV2/9-hSyn-DIO-jGCaMP7s-WPRE-pA was injected into STN.

To optogenetically manipulate STN excitatory neurons, 50 nl of AAV2/9-hEF1a-DIO-hChR2(H134R)-mCherry-WPRE-pA or AAV2/9-CaMKII-ChETA–EGFP-ER2-WPRE-pA, AAV2/9-Ef1a-DIO-mCherry-WPRE-pA, AAV2/8-CAG-DIO-GtACR1-p2A-EGFP, or AAV2/9-hEf1a-DIO-EGFP-WPRE-pA was injected into STN. To optogenetically manipulate STN^EP^ and STN^RtTg^ neurons, 30 and 60 nl of retroAAV-CaMKIIα-CRE-WPRE-pA were injected into the EP and RtTg, respectively; 50 nl of AAV2/9-hEF1a-DIO-hChR2(H134R)-mCherry-WPRE-pA, AAV2/9-Ef1a-DIO-mCherry-WPRE-pA, AAV2/8-CAG-DIO-GtACR1-p2A-EGFP, or AAV2/9-hEf1a-DIO-EGFP-WPRE-pA was injected into STN. AAV2/9-CaMKII-ChETA–EGFP-ER2-WPRE-pA was only performed in recording test.

To label whole-brain inputs to STN^EP^ neurons, 30 nl of retroAAV-CaMKIIα-CRE-WPRE-pA was injected into EP, 25 nl of AAV2/9-Ef1a-DIO-His-EGFP-2a-TVA-WPRE-pA mixed with AAV2/9-Ef1a-DIO-RVG-WPRE-pA was injected into the STN. Twenty-one days later, 100 nl of RV-EnvA-DG-mCherry was injected into the STN. To label whole-brain inputs to STN^RtTg^ neurons, 30 nl of retroAAV-CaMKIIα-CRE-WPRE-pA was injected into RtTg, 25 nl of AAV2/9-Ef1a-DIO-His-EGFP-2a-TVA-WPRE-pA mixed with AAV2/9-Ef1a-DIO-RVG-WPRE-pA was injected into the STN. Twenty-one days later, 100 nl of RV-EnvA-DG-mCherry was injected into the STN.

For mice undergoing surgery, anesthesia was induced under 4% and maintained under ~1.5% isoflurane. The left dorsolateral striatum was injected at two sites (+1.0 mm AP, +2.1 mm ML, −3.4 mm DV; +0.3 mm AP, +2.3 mm ML, −3.3 mm DV) using a 33-gauge needle, with 2 μl of 6-OHDA-bromide (to render mice parkinsonian) or normal saline (for control animals) per site. 6-OHDA or saline was injected at a rate of 0.2 μl/min, after which the injection cannula was left in place for 10 min before being withdrawn and the scalp being sutured. Parkinsonian animals were monitored closely for 1 week following surgery with daily saline injections and nutritional supplements (jelly and forage/trail mix). For subsequent optical manipulations or fiber photometry recording, viral vectors were delivered via pulled glass pipettes (5 ml, Drummond) using an automated injection system (Stoelting) 15 to 21 days after initial surgeries. Photometry and optogenetic experiments were performed after stabilization. Optical fiber-ferrule assemblies were implanted and secured in place with dental cement (Metabond) and dental acrylic (Ortho-Jet).

### Behavior

Apomorphine tests were applied 14 days after the operation for preliminary screening of the mice. To be specific, parkinsonian mice were injected with apomorphine (15 mg/kg) and immediately monitored in the open field for 15 min. Only the mice that had dyskinetic behavior or whose rotation times were above 7 times per minute would continue with the virus injection. Daily administration of levodopa was commenced 3 weeks after the virus injection. All mice were habituated to the open field (clear acrylic cylinders, 30-cm diameter) for 30 min 1 to 2 days before behavioral sessions. The mice were monitored via two cameras, one directly above (to capture overall movement) and one in front of the chamber (to capture fine motor behaviors). LID was scored by the same blinded rater. AIMs in axial (Ax), limb (Li), and orofacial body segments (Ol) were rated on a frequency scale during every 1-min observation. A score of 0 represents no abnormal movement, and a score of 4 represents continuous and uninterrupted dyskinetic movements. A score of 1 means occasional dyskinesia displaying for <50% of the observation time, and sustained dyskinesia displaying for >50% of the observation time was defined as a score of 2. A score of 3 indicates continuous and interruptable dyskinesia, while a score of 4 means continuous dyskinesia not interruptible by external stimuli. Notably, we separated turning from torsion and rotation. The torsion was axial dystonia accompanied by lifting of the forelimb from the ground and usually occurred during the apparent phase of LID, as reflected by the Ax score, whereas rotation was defined as turning with a larger radius during locomotion.

Video-tracking software (Noldus Ethovision) was used to quantify locomotor activity, including rotations, distance traveled, and velocity. The locomotion states were determined on the basis of the level of locomotor activity. Ambulation was defined as periods in which the velocity of the animal’s center point averaged more than 2 cm/s for a duration of at least 0.5 s. Immobility was defined as continuous periods of time during which the average pixel change across the entire video image was less than 2% for at least 1 s. Fine movement encompassed any type of movement that did not fall under the categories of ambulation or immobility.

### Pharmacology

6-OHDA (Sigma-Aldrich) for intrastriatal dopamine depletions was prepared at 2.5 mg/ml in a normal saline solution. Levodopa (Sigma-Aldrich) was always coadministered with benserazide (Sigma-Aldrich) and prepared in a normal saline solution. Levodopa was given via intraperitoneal injection 5 to 7 days per week, thereby mimicking the chronic levodopa treatment in a dopamine-depletion state. Animals received levodopa (6 to 10 mg/kg) and benserazide (3 to 5 mg/kg) to induce LID. To induce the medicine “on” and subdyskinesia state, levodopa (1 to 3 mg/kg) and benserazide (0.5 to 1.5 mg/kg) were used.

### Optogenetic manipulations

Before optical stimulation experiments, animals were habituated to tethering with lightweight patch cables (components: Inper Ltd.) coupled to an optical commutator (Doric Lenses) in the open field for 30 min, over 1 to 2 days. For all optogenetic manipulations, each mouse underwent behavioral testing in different states on separate days, including dyskinetic, subdyskinetic, and parkinsonian conditions. On each testing day, mice were first allowed to habituate to the open field for 15 min, followed by five optogenetic sessions, each consisting of a 60-s “light on” period and a 2-min “light off” period. TTL-controlled blue laser light (488 nm, Shanghai Laser and Optics Century) was delivered in pulse trains (10 ms, 14 Hz, controlled with Master 8, A.M.P.I.) for all optical reactivation experiments, or green laser light (532 nm, Shanghai Laser and Optics Century) was delivered continuously for all optical inhibition experiments. Animals were manually scored for dyskinetic behavior by raters blinded to the manipulation [e.g., ChR2 versus enhanced yellow fluorescent protein (eYFP)] during the baseline, light on/off, and post period. Video tracking software (EthoVision XT, Noldus) was used to measure movement. For optical reactivation experiments, animals did not receive levodopa for at least the previous 24 hours. For optical inhibition experiments, levodopa was administered 30 min before testing to capture maximal dyskinesia. At the end of the experimental procedures, animals were returned to their home cages.

### In vivo recordings with optogenetic stimulation

For optogenetic tagging of STN neurons, blue laser light (6 to 8 mW at fiber tip) was applied to the fiber of the optrode in free-moving, Vglut2-Cre mice. To identify a unit as ChETA expressing, we required that the unit was significantly activated by laser stimulation, the latency of laser-evoked spikes was <10 ms, and the Pearson’s correlation coefficient between waveforms of laser-evoked spikes and spontaneous spikes was >0.95. To identify the best stimulation frequency, we delivered 80 trials of 30-s laser stimulation at gradient frequencies (5, 20, 50, and 120 Hz), with a 30-s intertrial interval. We used the signed-rank test to compare the averaged firing rates between the spikes in a 1-s period before laser onset and the spikes in a 1-s period after laser onset at four frequencies. We computed the mean and SD for the spikes in a 1-s period before laser onset and defined a threshold as mean + 3 × SD. For each stimulation frequency, those units whose spike number in a 1-s period after laser onset was above the threshold were defined as “responding neurons,” and the others were “non-responding neurons.” We calculated “extra spikes” of each unit by subtracting the prelaser spike number from the laser-on spike number.

To verify the inhibitory effect of GtACR1, green laser light (6 to 8 mW at fiber tip, 60-s ON and 120-s OFF) was applied to the fiber of optrode in head-fixed, Vglut2-Cre mice for 20 trials. To quantify the degree of firing rate change, we computed a rate change index as (*R*_laser_on_ − *R*_laser_off_)/(*R*_laser_on_ + *R*_laser_off_), in which *R*_laser_on_ and *R*_laser_off_ represented responses for laser-on and laser-off trials, respectively. To determine the effect of laser stimulation on firing rate, we used Welch’s *t* test to compare the rate change index and 0. We used the signed-rank test to compare the spikes in a 60-s period during laser on versus the spikes in a 60-s period before laser onset (or after laser offset).

### Fiber photometry recording

An optical fiber (200-μm optical density, 0.37 numerical aperture, Inper) was implanted 100 μm above the viral injection site or over its viral expressed terminal site in the STN and secured with dental cement. To minimize photobleaching, photometry recordings were performed using an interval recording mode. Fluorescence signals were recorded using a Fiber Photometry system (Inper) at a 120-s light-on /180-s light-off cycle. Each recording day began with a 15-min baseline epoch (3 epochs), followed by recordings of 150 min in the dyskinetic condition (30 epochs) and 90 min in the subdyskinetic and parkinsonian conditions (18 epochs per condition). The 488- and 410-nm excitation light sources were given alternately, and 410 nm was used as the control. We corrected signals using 410-nm reference normalization, exponential fitting, and motion correction. The power of 488-nm lights at the exit end of the fiber ranged from 50 to 55 μW, and that for 410-nm lights ranged from 15 to 20 μW. The laser power remained stable throughout each experiment, and photobleaching was modest (~5 to 10% over 180 min).

### Fiber photometry data analysis

We used an interval recording strategy with 2-min recording epochs interleaved with 3-min intervals, including only signals from the recording epochs to obtain a de-interred signal. To exclude potential interference of the signal by the fluorescent switch, we specifically extracted the middle 1-min segment of each recording epoch. To reduce the photobleaching effect caused by long-term recording, we fit an exponential to the 410-nm control signal and then subtracted it from the 488-nm signal to yield a baseline-corrected signal. The 410-nm bleach-corrected signal was then subtracted from the baseline-corrected 488-nm signal to yield a motion-corrected signal. This approach ensured minimal interference and provided a representative analysis of the signals.

We calculated the mean value of the preinjection signal (*F*_0_) as a baseline representing minimal motion. The corrected 488-nm signal was used to determine Δ*F*/*F*, computed as (*F* − *F*_0_)/*F*_0_. To capture episode-related changes, we normalized the Δ*F*/*F* value by subtracting it from the mean Δ*F*/*F* of the preceding epoch, highlighting the specific impact of each episode. Heatmaps were generated for dynamic calcium signals at the recording epoch aligned to dyskinesia episodes, and *z*-score Δ*F*/*F* was calculated to standardize the data across mice. The corresponding AIM score and rotation counts were evaluated concurrently. Data were extracted, processed, and analyzed using custom MATLAB (The Mathworks) scripts. Mice with off-target fiber-tip location were excluded from the analysis.

### Anterograde and retrograde tracing

For whole brain mapping the outputs of STN, AAV9-hSyn-FLEX-tdTomato-T2A-Synaptophysin-EGFP mixed with AAV-CaMKIIα-Cre were injected in STN (−1.8 mm AP, +1.6 mm ML, −4.6 mm DV). To identify the location and overlap of STN^EP^ and STN^RtTg^, retroAAV-EGFP and retroAAV-mCherry were injected in EP (−1.1 mm AP, +2.0 mm ML, −4.45 mm DV) and RtTg (−4.17 mm AP, +0.3 mm ML, −5.15 mm DV), respectively.

For rabies virus tracing, retroAAV-CaMKIIα-Cre was injected in the STN output targets (EP or RtTg) to express Cre recombinase in STN^EP^ or STN^RtTg^ neurons. Meanwhile, two Cre-dependent viruses, AAV-DIO-His-EGFP-2A-TVA and AAV-DIO-RVG, were mixed at a volume ratio of 1:2 and injected into the STN of wild-type mice with a volume of 60 nl. After 3 weeks of recovery and AAV expression, 100 nl of RV-EnvA-DG-dsRed was injected into the same location. The injection of rabies virus was performed in a biosafety level-2 environment. After 1 week of rabies virus infection and transsynaptic spread, the animals were euthanized and proceeded to histology.

### Histology, microscopy, and cell counting

At the end of each experiment, mice were deeply anesthetized with isoflurane and transcardially perfused with phosphate-buffered saline (PBS) followed by ice-cold 4% paraformaldehyde (PFA) and then transferred in 30% sucrose at 4°C for cryoprotection. The brain was then cut into 30-μm coronal sections on a freezing microtome (Leica) and was blocked in 5% goat serum, 0.4% Triton X-100, and 3% bovine serum albumin in 1 × PBS for 2 hours. We applied the c-Fos antibody (1:250; no. 2250S; Cell Signaling Technology) or TH antibody (1:500; no. ab112; Abcam) overnight at 4°C followed by 3 hours at room temperature the next day. Goat anti-rabbit 594 (1:1000; no. A11012; Thermo Fisher Scientific) or 647 (1:1000; catalog no. A21235; Invitrogen), goat anti-mouse 488 (1:1000; no. A11008; Invitrogen), and 4′,6-diamidino-2-phenylindole (DAPI) (1:10,000 of 5 mg/ml) were applied for 2 hours at room temperature. Confocal images were obtained with the Leica STED sp8 system.

### Single-molecular fluorescent in situ hybridization

The single-molecular fluorescent in situ hybridization using the RNAScope Multiplex Reagent Kits (ACDBio, United States) was performed on the fresh-frozen sections from the mice euthanized 40 min after the cessation of behavioral stimulation. Briefly, we cut 20-μm frozen sections and air-dried them inside the cryostat for 20 min. Frozen sections were fixed in ice-cold 4% PFA for 15 min followed by dehydration in ethanol series. The slides were treated with H_2_O_2_ for 10 min and washed in PBS. Protease III was added and incubated for 20 min at room temperature. CaMKIIα and Vglut2 probes were diluted at a 1:50 ratio with probe diluent. Sections were incubated with the probes for 2.5 hours at 40°C inside the HybEZ humidified incubator, then rinsed with ACD wash buffer. Slides were sequentially incubated in reagents AMP1-FL and AMP2-FL for 30 min, and then AMP3-FL for 15 min, followed by DAPI. Image acquisition was obtained using the Leica Sp8 confocal system.

### Statistical analysis

Statistical comparisons were conducted using the significance tests mentioned in the main text and performed in Prism. Statistical significance was defined as *P* < 0.05 unless otherwise stated. Data were tested for normality with the Shapiro-Wilk test, and parametric/nonparametric tests were used as appropriate. Pairwise comparisons were made with paired two-tailed *t* tests or signed-rank test (two tailed) using Prism. Differences between two groups were compared by Welch’s *t* test (two tailed) for normally distributed variables and Mann-Whitney test (two tailed) for non-normally distributed variables. One-way, two-way, or repeated measures analyses of variance (ANOVAs) with Bonferroni post hoc test were performed in Prism to make comparisons across multiple groups. We interrogated correlations using Pearson’s *r* correlation and Pearson’s partial correlation coefficients. Detailed statistical analyses are summarized in tables S1 to S3. n.s, *P* > 0.05; *P < 0.05; **P < 0.01; ***P < 0.001.
