# Supplementary material for: Distinct contributions of two subpopulations of subthalamic neurons to levodopa-induced dyskinesia
Source: Sci Adv. 2026 Jul 3;12(27):eaed2912. doi: 10.1126/sciadv.aed2912 (PMC13330827; doi:10.1126/sciadv.aed2912)
Supplement: Supplementary file 1 — Figs. S1 to S10 Legends for tables S1 to S3 Legends for movies S1 to S6 [file sciadv.aed2912_sm.pdf]

Science Advances
AAAS

Supplementary Materials for

Distinct contributions of two subpopulations of subthalamic neurons to levodopa-induced dyskinesia

Bo Shen *et al.*

Corresponding author: Haishan Yao, haishanyao@ion.ac.cn; Jianjun Wu, wujianjun@fudan.edu.cn; Jian Wang, wangjian_hs@fudan.edu.cn

Sci. Adv. **12**, eaed2912 (2026)
DOI: 10.1126/sciadv.aed2912

The PDF file includes:

Figs. S1 to S10
Legends for tables S1 to S3
Legends for movies S1 to S6

Other Supplementary Material for this manuscript includes the following:

Tables S1 to S3
Movies S1 to S6

Supplementary Figures and Figure Legends

Fig. S1. Caudal STN outputs to distinct brainstem regions. Related to Fig. 1.

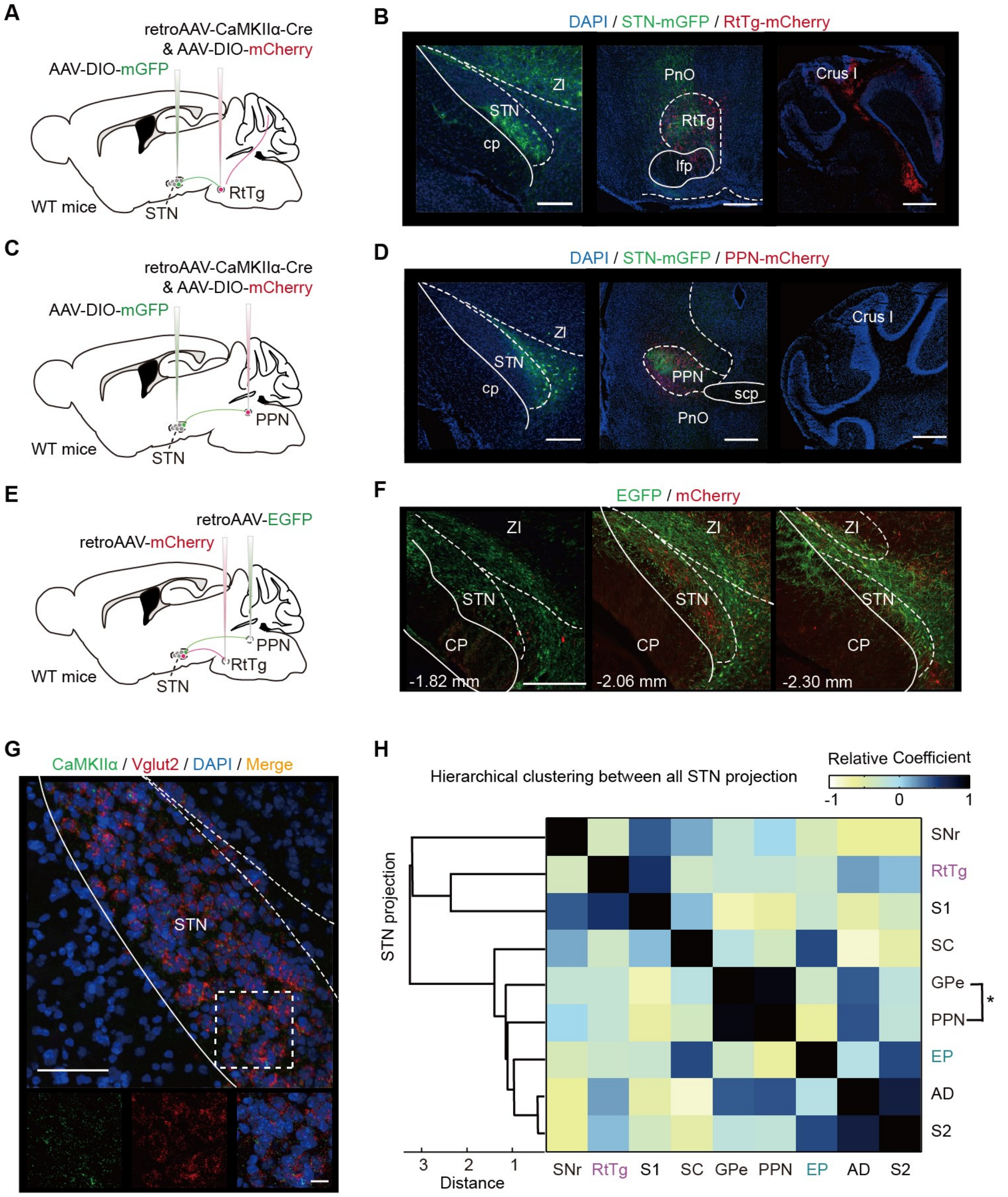

(**A** and **C**) Viral strategies for the localization of the RtTg (**A**) or PPN (**C**) projecting STN subpopulation. (**B** and **D**) Coronal section showing the localization of two distinct pontine projecting STN subpopulations (left), mGFP^{+} axon terminals of STN neurons and the injection site with mCherry (middle), and the RtTg innervation of the cerebellum (right). Scale bar, 200 μm. (**E**) Viral strategy to simultaneously label STN^{RtTg} and STN^{PPN} neurons. (**F**) Representative images of the topological distribution of STN^{RtTg} and STN^{PPN} neurons along the anterior-posterior axis of the STN. STN^{PPN} crossed the STN boundary without a clear distribution trend. Scale bar, 200 μm. (**G**) The mRNA signals of both CaMKIIα and Vglut2 are widely expressed in the vast majority of STN

neurons for the dual labeling RNAscope experiments. Scale bar, 20 µm. (**H**) Heatmap showing hierarchical clustering of nine downstream target regions based on their projection intensity correlation patterns. Fluorescence intensities from STN projections to each target were quantified from n=5 mice. Data were Z-scored across targets for each mouse to ensure equal weighting, and Pearson's correlation coefficients were calculated between all pairs of target regions. Hierarchical clustering was performed using Pearson's correlation coefficient as the distance metric and Ward's method as the linkage criterion. The color scale indicates the degree of correlation (dark, high correlation; white, low correlation). The dendrogram to the left of the heatmap illustrates the clustering relationships among target regions. The cluster separating projections to EP and RtTg from other targets suggests distinct projection patterns. The high correlation between GPe and PPN may indicate collateral projections from the same STN neurons. Statistics detailed in Table S3.

Fig. S2. Different kinetic states after levodopa administration. Related to Figs. 2 and 3.

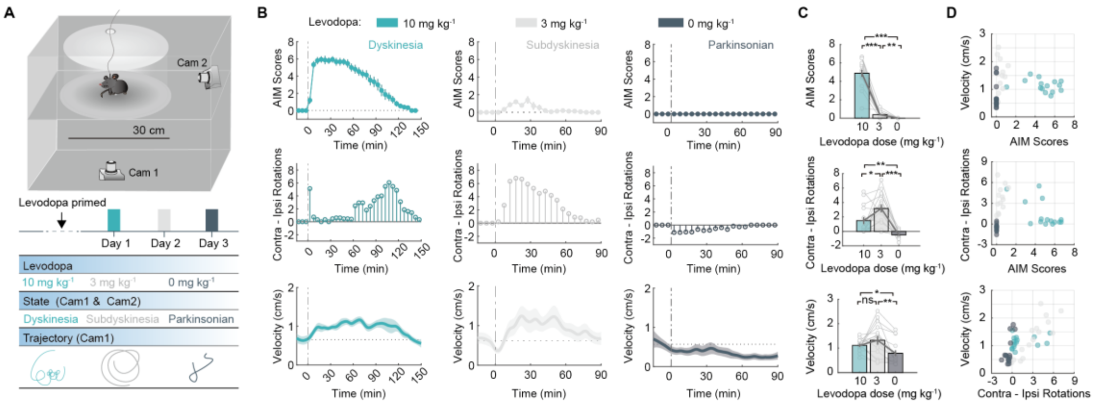

(**A**) Mice were placed in a circular locomotion arena equipped with two orthogonally positioned cameras. Levodopa-primed mice, which were administered with different doses of levodopa for three consecutive days, were expected to develop kinetic states of dyskinesia, subdyskinesia, or parkinsonian. The movement trajectories were captured by the camera. (**B**) Dynamics of AIM scores, rotation bias (contra- minus ipsi-lesional rotations per minute), and velocity for mice in different kinetic states. (**C**) AIM scores, rotation rates, and velocity after levodopa administration at different doses. (n = 15 mice. ns, nonsignificant, **P* < 0.05, ***P* < 0.01, and ****P* < 0.001; two-tailed Paired t-test) (**D**) Correlation among AIM scores, rotation rates, and velocity in various states. All data are shown as mean ± s.e.m. Statistics detailed in Table S3.

Fig. S3. Effect of optogenetic activation or inactivation on the firing rates of STN neurons in vivo. Related to Fig. 4 and Fig. 6.

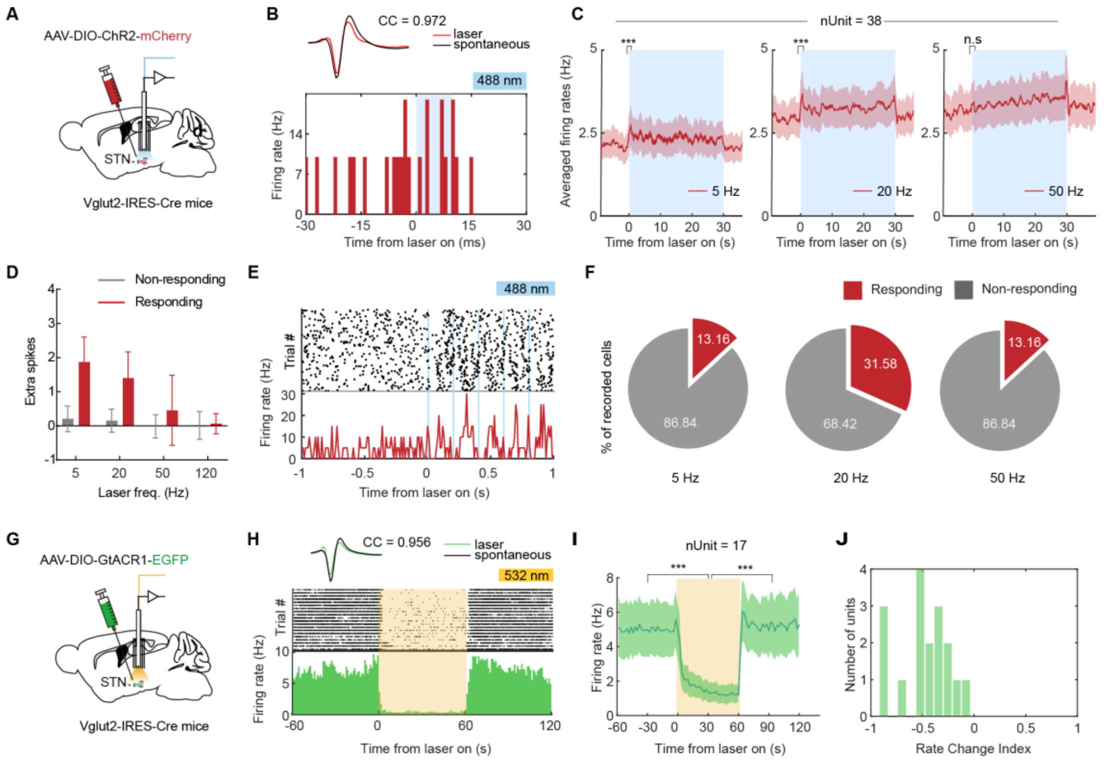

(**A**) Schematic showing virus injection and optrode array implantation in STN to test the effect of activating ChR2. (**B**) Response of an example STN unit. Top, average spike waveforms (black, spontaneous; red, light-evoke). Bottom, peri-stim firing rate histogram (1 ms/bin) showing the response to 10-ms laser. Blue shading, laser stimulation. CC, Pearson's correlation coefficient between spontaneous and light-evoked spike waveforms. (**C**) Histogram of averaged firing rates of STN neurons in response to 5 Hz, 20 Hz, and 50 Hz laser stimulation. n = 38 units. ***P = 0.0002 for 5 Hz, ***P <0.001 for 20 Hz, P = 0.823 for 50 Hz; Sign-rank test, bin size = 100 ms. (**D**) Comparison of laser-evoked extra spikes between responding and non-responding neurons for each stimulation frequency. n = 38 units. ***P < 0.001 for 5 Hz, ***P <0.001 for 20 Hz, P = 0.2211 for 50 Hz, P = 0.6842 for 120 Hz; Mann-Whitney test. (**E**) Raster plot (top) and peri-stimulus time histogram (bottom) of an example putative ChR2^{+} STN neuron. Bin size = 10 ms. Blue, 10-ms laser stimulation at 5 Hz. (**F**) Pie graphs depicting percentages of STN neurons in response to stimulation at a range of frequencies (n = 38 units), responding units were optically identified by their spike number in a 1s period after laser onset was above the threshold (mean + 3×SD). (**G**) Schematic showing virus injection and optrode recording in the STN to test the effect of activating GtACR1. (**H**) Raster plot (top) and peri-stimulus time histogram (bottom) of an example STN neuron from a Vglut2-Cre mouse, in which AAV-DIO-GtACR1-EGFP was injected into STN. Binsize = 1000 ms. (**I**) Firing rates before, during, and after laser stimulation. Activation of GtACR1 for 60 s could effectively reduce the firing rates of STN excitatory neurons. n = 17 units. ***P <0.001; Sign-rank test. (**J**) Distribution of rate change indexes of STN neurons stimulated with 532 nm laser. n = 17 units. ***P < 0.001; Welch's t-test. Rate change index was computed as (R_{laser_on} − R_{laser_off}) / (R_{laser_on} + R_{laser_off}), in which R_{laser_on} and R_{laser_off} represented responses for laser-on and laser-off

trials, respectively. All data are shown as mean ± s.e.m. Statistics detailed in Table S3.

Fig. S4. Effect of activating different populations of STN neurons on AIM score subdomains. Related to Fig. 4 and Fig. 6.

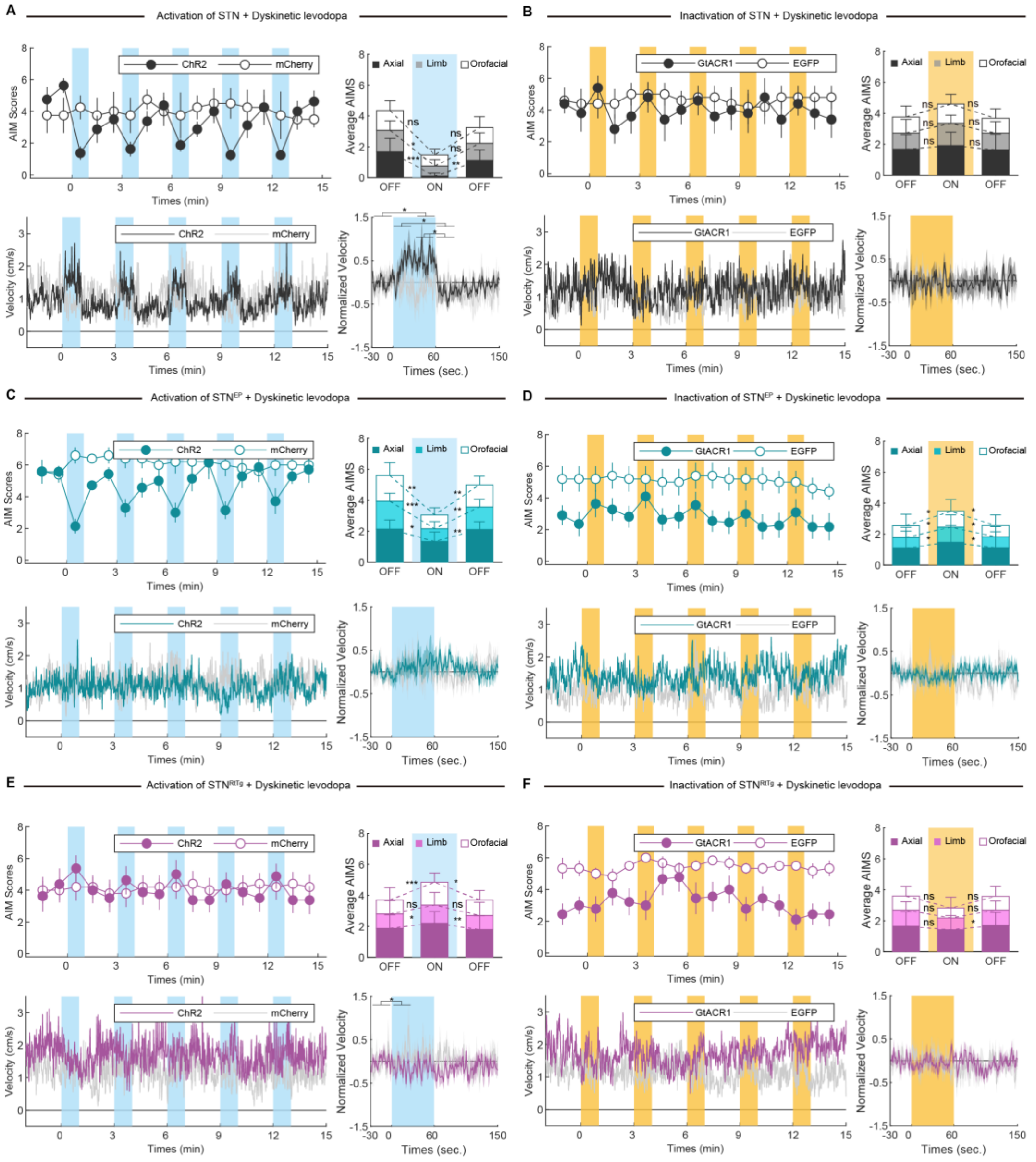

(**A** and **B**) Top: Effect of activation (**A**) and inactivation (**B**) of STN excitatory neurons on average AIM scores (left) and subdomains (right) during light-on and light-off epochs in the LID state. (n = 8 mice for ChR2 and mCherry, respectively; n = 5 mice for GtACR1 and EGFP, respectively. ns, nonsignificant, **P* < 0.05, ***P* < 0.01, and ****P* < 0.001; two-tailed paired t-test). Bottom: Effect of activation (**A**) and inactivation (**B**) of STN excitatory neurons on velocity. (n = 8 mice for ChR2 and mCherry, respectively; n = 5 mice for GtACR1 and EGFP, respectively. ns, nonsignificant, **P* < 0.05; Wilcoxon rank-sum test). Activation of STN excitatory neurons significantly decreased average axial and limb scores during the stimulation epoch, but did not change average orofacial scores. Inactivation of STN excitatory neurons did not have a significant effect. (**C** and **D**) Top: Effect of activation (**C**) and inactivation (**D**) of STN^{EP} neurons on average AIM scores (left) and subdomains (right) during light-on and light-off epochs in the LID state. (n = 7 mice for ChR2 and

n = 5 mice for mCherry; n = 11 mice for GtACR1 and n = 5 mice for EGFP. *$P < 0.05$, **$P < 0.01$, and ***$P < 0.001$; two-tailed paired t-test). Bottom: Effect of activation (**C**) and inactivation (**D**) of STN^{EP} neurons on velocity. (n = 7 mice for ChR2 and n = 5 mice for mCherry; n = 11 mice for GtACR1 and n = 5 mice for EGFP. *$P < 0.05$; Wilcoxon rank-sum test). Activation of STN^{EP} neurons significantly decreased average axial, limb, and orofacial scores during the stimulation period, while inactivation of STN^{EP} neurons had an opposite effect. (**E** and **F**) Top: Effect of activation (**E**) and inactivation (**F**) of STN^{RtTg} neurons on average AIM scores (left) and subdomains (right) during light-on and light-off epochs in the LID state. (n = 8 mice for ChR2 and n = 5 mice for mCherry; n = 9 mice for GtACR1 and n = 6 mice for EGFP. ns, nonsignificant, *$P < 0.05$, **$P < 0.01$, and ***$P < 0.001$; two-tailed paired t-test). Bottom: Effect of activation (**E**) and inactivation (**F**) of STN^{RtTg} neurons on velocity. (n = 8 mice for ChR2 and n = 5 mice for mCherry; n = 9 mice for GtACR1 and n = 6 mice for EGFP. ns, nonsignificant and *$P < 0.05$; Wilcoxon rank-sum test). Activation of STN^{RtTg} neurons had an opposite effect compared to activation of STN^{EP} neurons, with increasing average axial and orofacial scores during the stimulation period. Inactivation of STN^{RtTg} neurons did not have a significant effect. All data are presented as mean ± s.e.m. Statistics detailed in Table S3.

Fig. S5. Optogenetic manipulation of STN neurons regulates motor behavior in the subdyskinesia state. Related to Fig. 4 and Fig. 6.

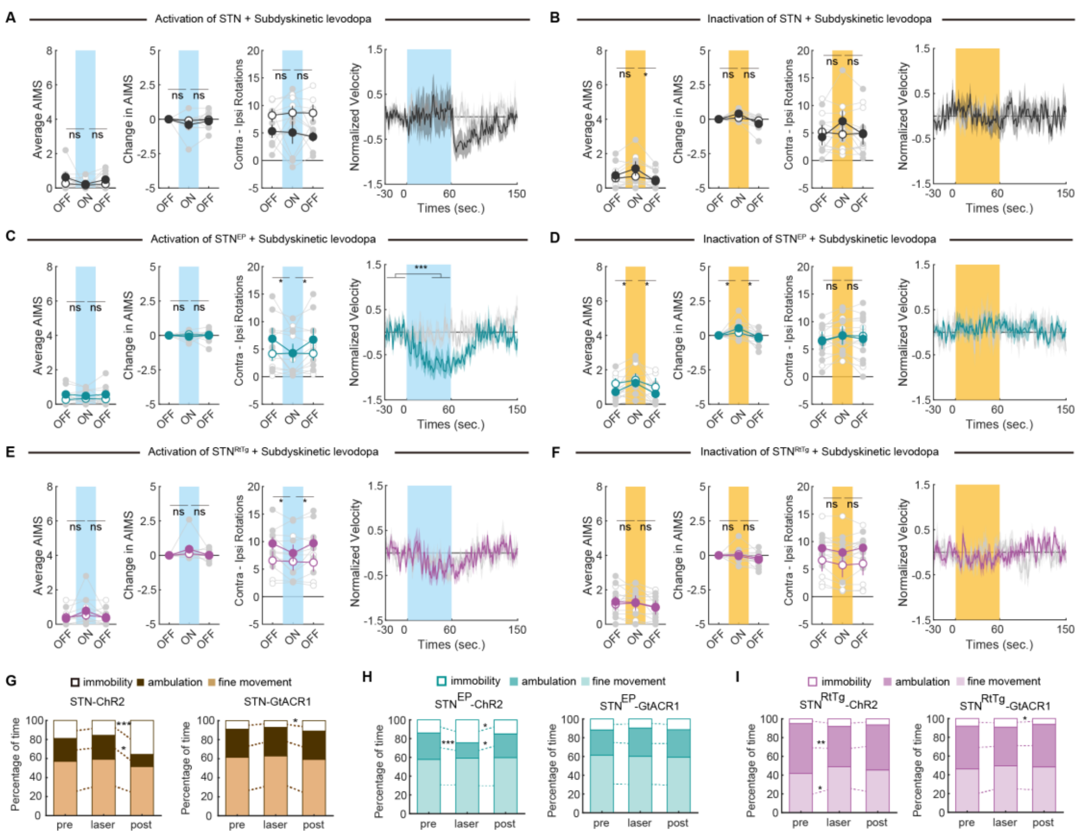

(**A** and **B**) Effect of activating (**A**) and inactivating (**B**) STN excitatory neurons on average AIM scores during light-on and light-off epochs, change in AIM scores, rotation bias, and normalized velocity in the subdyskinesia state (n = 7 mice for ChR2 and n = 6 mice for mCherry; n = 6 mice for GtACR1 and n = 8 mice for EGFP. ns, nonsignificant and **P* < 0.05; two-tailed Paired t-test and Wilcoxon rank-sum test). - (**C** and **D**) Effect of activating (**C**) and inactivating (**D**) STN^{EP} neurons on average AIM scores during light-on and light-off epochs, change in AIM scores, rotation bias, and normalized velocity in the subdyskinesia state. (n = 7 mice for ChR2 and n = 5 mice for mCherry; n = 12 mice for GtACR1 and n = 4 mice for EGFP. ns, nonsignificant, **P* < 0.05, ****P* < 0.001; two-tailed Paired t-test and Wilcoxon rank-sum test). (**E** and **F**) Effect of activating (**E**) and inactivating (**F**) STN^{RtTg} neurons on average AIM scores during light-on and light-off epochs, change in AIM scores, rotation bias, and normalized velocity in the subdyskinesia state. (n = 9 mice for ChR2 and n = 6 mice for mCherry; n = 9 mice for GtACR1 and n = 6 mice for EGFP. ns, nonsignificant and **P* < 0.05; two-tailed Paired t-test and Wilcoxon rank-sum test). (**G** to **I**) Motor behavior before, during, and after activation (inactivation) of STN neurons in the subdyskinesia state. (n = 7 mice for STN-ChR2 and n = 7 mice for STN-GtACR1; n = 8 mice for STN^{EP}-ChR2 and n = 12 mice for STN^{EP}-GtACR1; n = 8 mice for STN^{RtTg}-ChR2 and n = 9 mice for STN^{RtTg}-GtACR1. ns, nonsignificant, **P* < 0.05, **P < 0.01, and ****P* < 0.001; two tailed Sign-rank test). All data are shown as mean ± s.e.m. Statistics detailed in Table S3.

Fig. S6. Optogenetic activation of STN neurons influences locomotion in Parkinsonian mice. Related to Fig. 4 and Fig. 6.

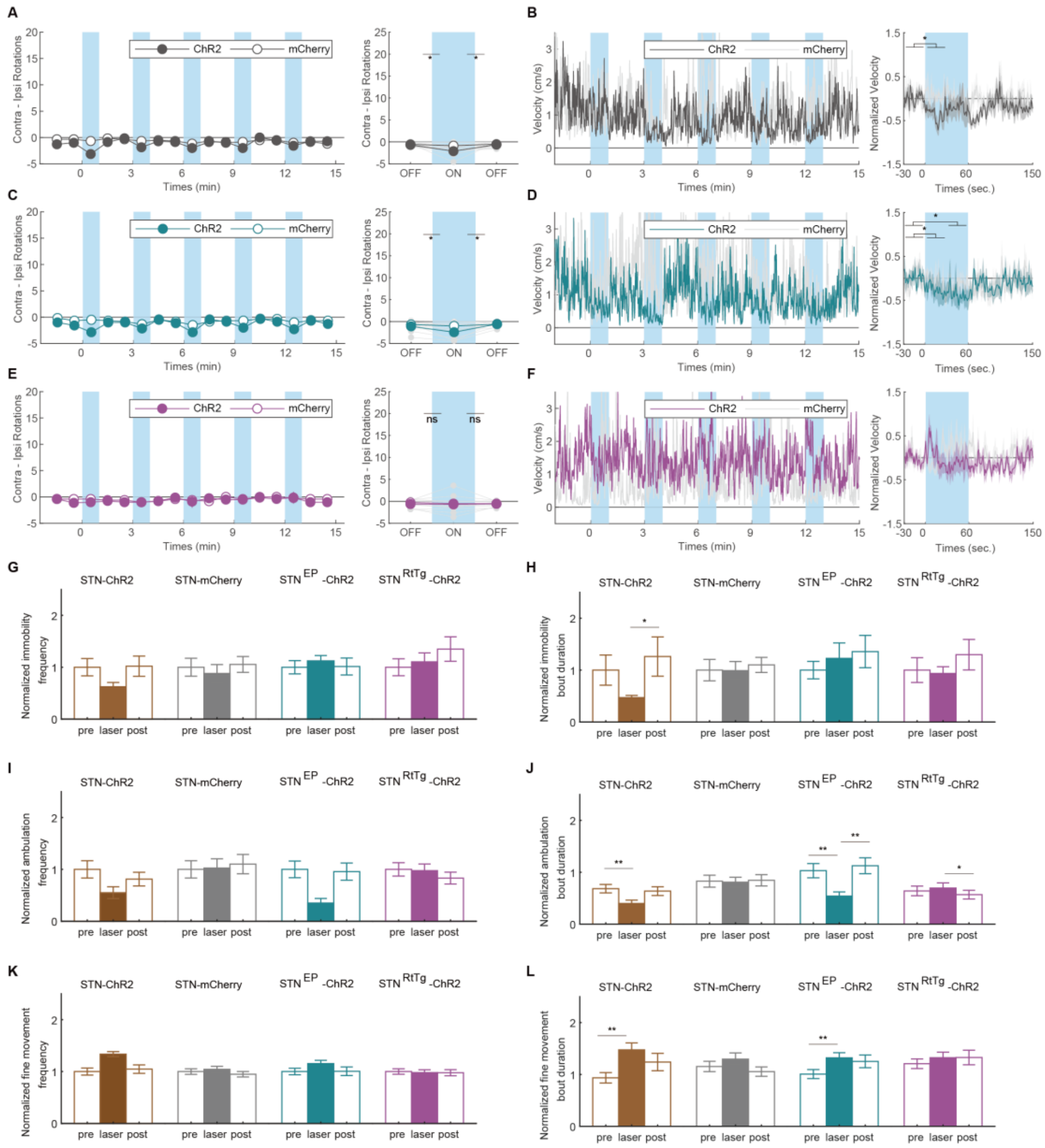

(**A** and **B**) Effect of activating STN excitatory neurons on rotation bias (**A**) and normalized velocity (**B**). (n = 7 mice for ChR2 and n = 6 mice for mCherry; ns, nonsignificant and $*P < 0.05$; two-tailed Paired t-testand Wilcoxon rank-sum test). (**C** and **D**) Effect of activating $\mathrm{STN}^{\mathrm{EP}}$ neurons on rotation bias (**C**) and normalized velocity (**D**). (n = 7 mice for ChR2 and n = 6 mice for mCherry; ns, nonsignificant and $*P < 0.05$; two-tailed Paired t-testand Wilcoxon rank-sum test . (**E** and **F**) Effect of activating $\mathrm{STN}^{\mathrm{RtTg}}$ neurons on rotation bias (**E**) and normalized velocity (**F**). (n = 8 mice for ChR2 and n = 5 mice for mCherry; ns, nonsignificant; two-tailed Paired t-test and Wilcoxon rank-sum test. (**G** to **L**) Effect of laser stimulation on the frequency of immobility (**G**), duration of immobility bouts (**H**), frequency of ambulation (**I**), ambulation bout duration (**J**), frequency of fine movement (**K**), fine-movement bout duration (**L**) in STN-ChR2 (yellow bars), STN-mCherry (gray bars), $\mathrm{STN}^{\mathrm{EP}}$-ChR2 (blue bars) and $\mathrm{STN}^{\mathrm{RtTg}}$-ChR2 (red bars). n = 7 mice for STN-ChR2, n = 6 mice

for STN-mCherry, n =7 mice for STN^{EP}-ChR2, and n = 8 mice for STN^{RtTg}-ChR2. ns, nonsignificant, *$P < 0.05$, and **$P < 0.01$. All data are shown as mean ± s.e.m. Statistics detailed in Table S3.

Fig. S7. Optogenetic inactivation of STN neurons influences locomotion in Parkinsonian mice. Related to Fig. 4 and Fig. 6.

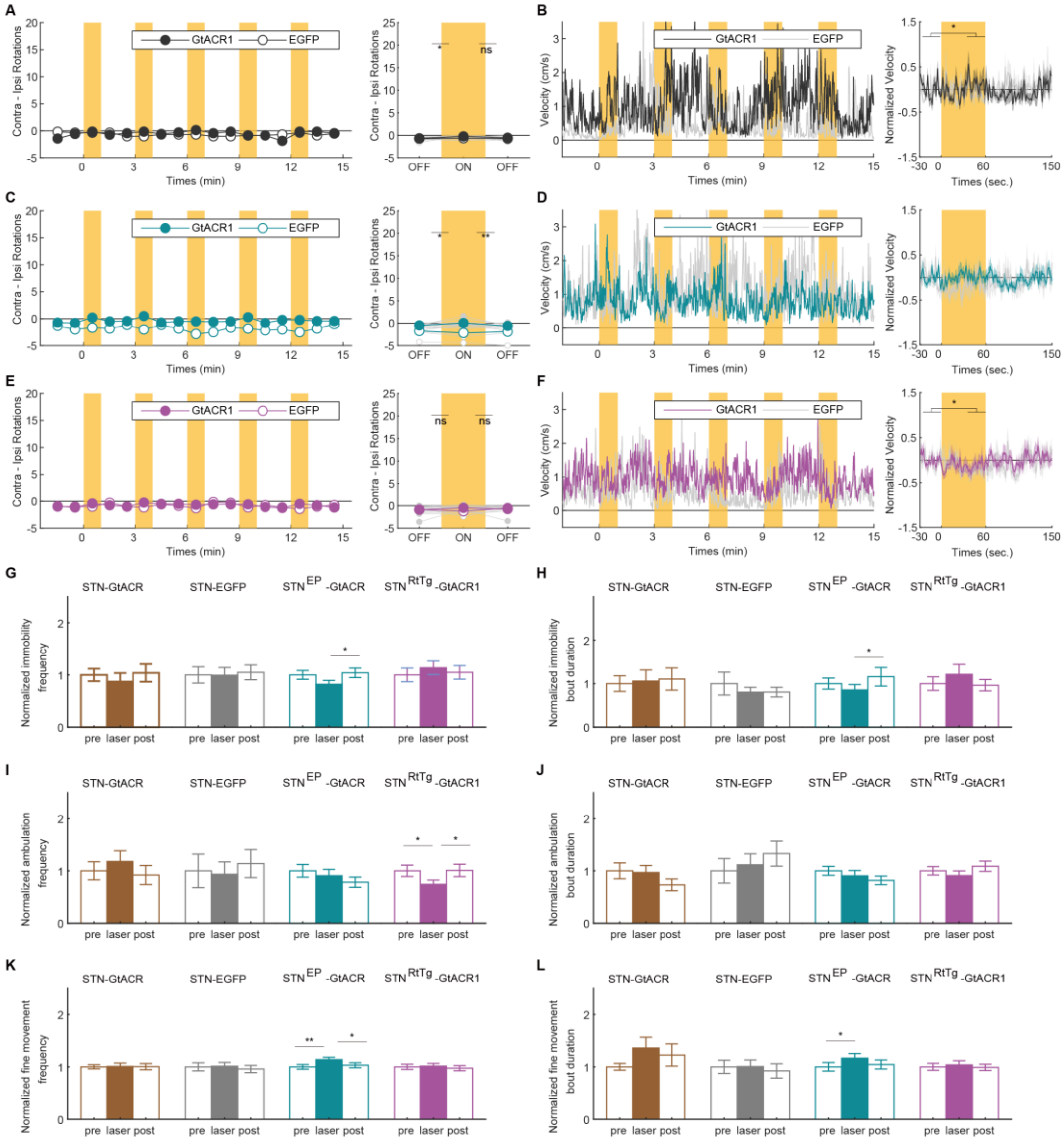

(**A** and **B**) Effect of inactivating STN excitatory neurons on rotation bias (**A**) and normalized velocity (**B**). (n = 7 mice for GtACR1 and n = 6 mice for EGFP; ns, nonsignificant and *$P < 0.05$; two-tailed Paired t-test and Wilcoxon rank-sum test). (**C** and **D**) Effect of inactivating STN^{EP} neurons on rotation bias (**C**) and normalized velocity (**D**). (n = 14 mice for GtACR1 and n = 6 mice for EGFP; ns, nonsignificant, *$P < 0.05$, and **$P < 0.01$; two-tailed Paired t-test and Wilcoxon rank-sum test). (**E** and **F**) Effect of inactivating STN^{RtTg} neurons on rotation bias (**E**) and normalized velocity (**F**). (n = 12 mice for GtACR1 and n = 7 mice for EGFP; ns, nonsignificant and *$P < 0.05$; two-tailed Paired t-test and Wilcoxon rank-sum test). (**G** to **L**) Effect of laser stimulation on the frequency of immobility (**G**), duration of immobility bouts (**H**), frequency of ambulation (**I**), ambulation bout duration (**J**), frequency of fine movement (**K**), fine-movement bout duration (**L**) in STN-GtACR1 (yellow bars), STN-EGFP (gray bars), STN^{EP}-GtACR1 (bule bars) and STN^{RtTg}-GtACR1 (red bars) mice. n = 7 mice for STN-GtACR1, n = 6 mice for STN-EGFP, n =14 mice for

STN^{EP}-GtACR1, and n = 12 mice for STN^{RtTg}-GtACR1. ns, nonsignificant, **P* < 0.05, and ***P* < 0.01. All data are shown as mean ± s.e.m. Statistics detailed in Table S3.

Fig. S8. Dyskinesia related STN^{EP} activity in different phases of dyskinesia. Related to Fig. 5.

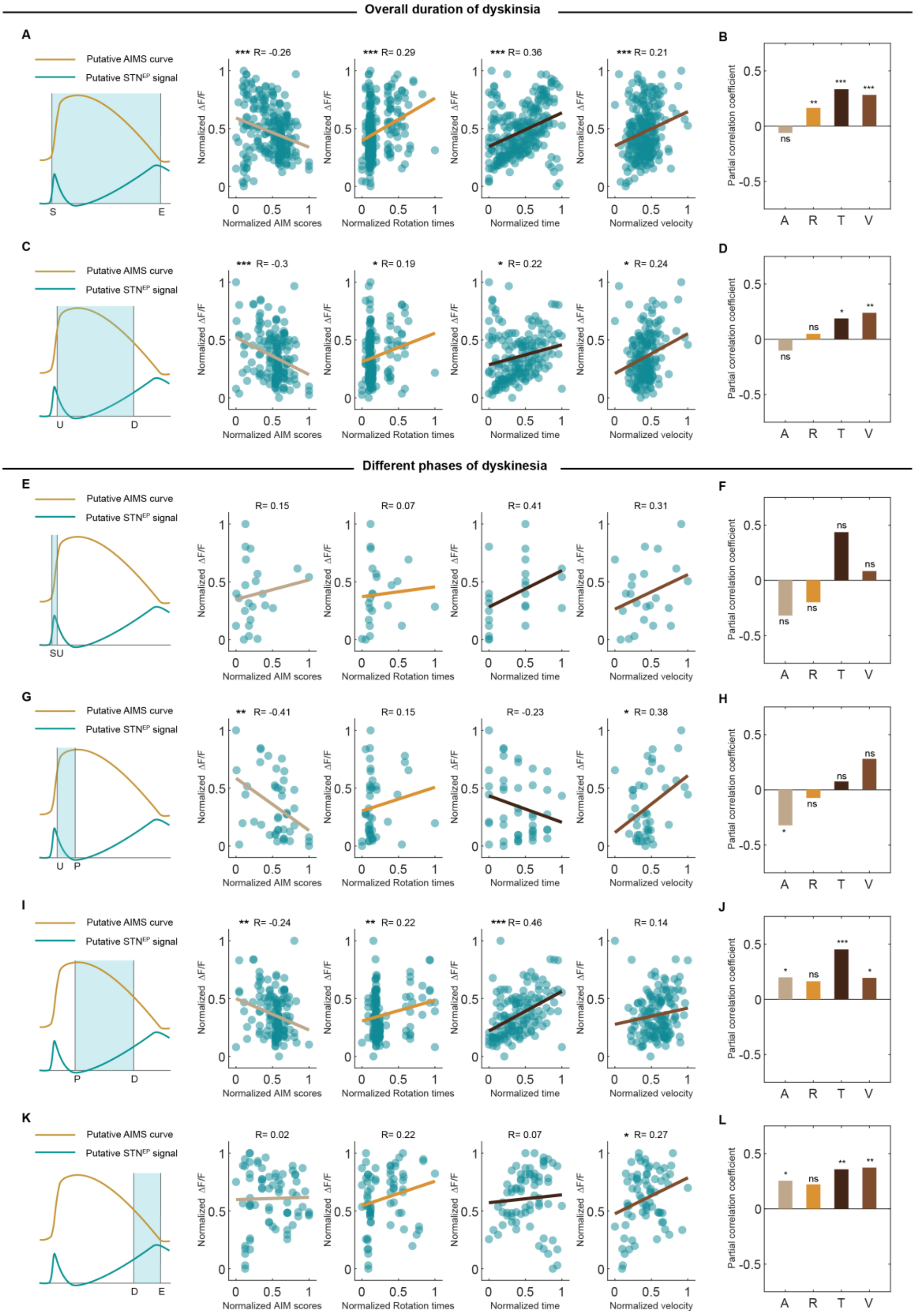

(**A** and **C**) During the entire duration of dyskinesia (**A**) or apparent phase, from AIM ramping up to ramping down (**C**). Left, observation episode. Right scatter plots, correlation between normalized

ΔF/F of STN^{EP} and AIM scores, rotation bias, velocity, or time after levodopa administration. (n =10 mice. ns, nonsignificant, **P* < 0.05, ***P* < 0.01, and ****P* < 0.001; Pearson's correlation). (**B** and **D**) Partial correlation coefficients between ΔF/F of STN^{EP} and each of the task variables, controlling for the remaining variables. (n =10 mice. ns, nonsignificant, **P* < 0.05, ***P* < 0.01, and ****P* < 0.001; Pearson partial correlations). (**E** to **L**) Correlation and Partial correlation coefficients between ΔF/F and each of the task variables during different phases of dyskinesia, from AIM start to ramping up (**E** and **F**), from AIM ramping up to peak (**G** and **H**), from AIM peak to ramping down (**I** and **J**), from AIM down to end (**K** and **L**). (n =10 mice. ns, nonsignificant, **P* < 0.05, ***P* < 0.01, and ****P* < 0.001; Pearson's correlation for (**E**, **G**, **I,** and **K**) and Pearson partial correlations for (**F**, **H**, **J,** and **L**)). All data are shown as mean ± s.e.m. Statistics detailed in Table S3.

Fig. S9. Dyskinesia related STN[RtTg] activity in different phases of dyskinesia. Related to Fig. 5.

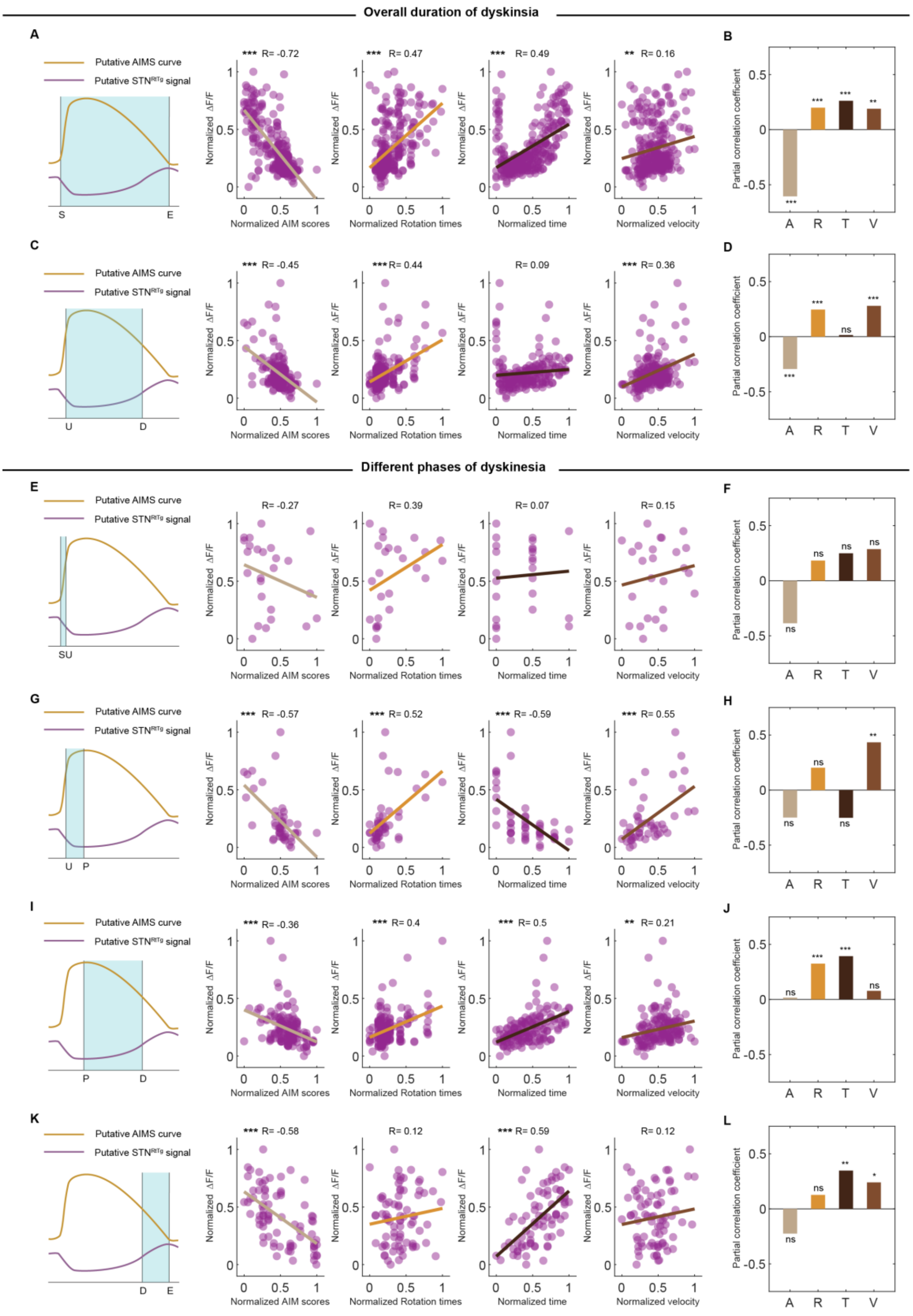

(**A** and **C**) During the entire duration of dyskinesia (**A**) or apparent phase, from AIM ramping up to ramping down (**C**). Left, observation episode. Right scatter plots, correlation between normalized

ΔF/F of STN^{RtTg} and AIM scores, rotation bias, velocity, and time after levodopa administration. (n =11 mice. ns, nonsignificant, ***P* < 0.01, and ****P* < 0.001; Pearson's correlation). (**B** and **D**) Partial correlation coefficients between ΔF/F of STN^{RtTg} and each of the task variables, controlling for the remaining variables. (n =11 mice. ns, nonsignificant and ****P* < 0.001; Pearson partial correlations). (**E** to **L**) Correlation and Partial correlation coefficients between ΔF/F and each of the task variables during different phases of dyskinesia, from AIM start to ramping up (**E** and **F**), from AIM ramping up to peak (**G** and **H**), from AIM peak to ramping down (**I** and **J**), from AIM down to end (**K** and **L**). (n =11 mice. ns, nonsignificant, **P* < 0.05, ***P* < 0.01, and ****P* < 0.001; Pearson's correlation for (**E**, **G**, **I,** and **K**) and Pearson partial correlations for (**F**, **H**, **J,** and **L**)). All data are shown as mean ± s.e.m. Statistics detailed in Table S3.

Fig. S10. Starter cells and control experiments for rabies virus tracing of inputs. Related to Fig. 7.

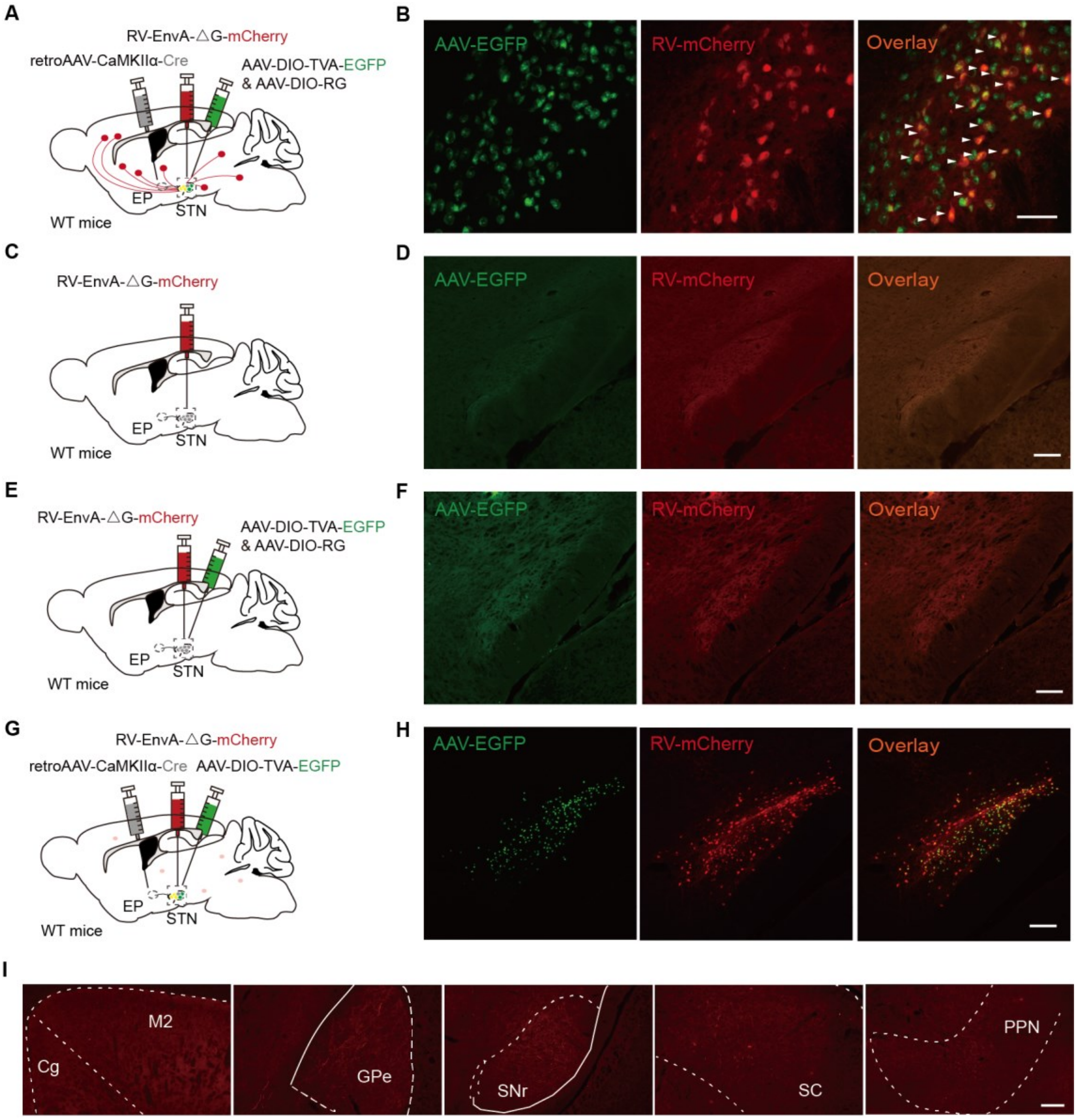

(**A** and **B**) Viral strategy to map inputs of STN^{EP} neurons (**A**) and examples of starter cells at the injection sites (**B**). (**C** and **D**) No neuron was labeled when RV-EnvA-mCherry alone was injected into the STN. (**E** and **F**) AAV helper virus and RV were injected into the brains of wild-type mice without retrograde transfection of Cre, and no neurons were labeled at the injection site. (**G** to **I**) Without AAV-DIO-RG, only neurons at the injection site were labeled. Scale bar, 50 μm for (**B**) and 200 μm for (**D**, **F**, and **H**). Statistics detailed in Table S3.

Tables S1–S3.

Due to their large width, these tables are provided as separate Excel files, including:

Table S1. Detailed strategy of virus injections. Related to Figures 1-7 and Figures S1-10.

Table S2. Summary of statistical analysis, related to Figures 1-7.

Table S3. Summary of statistical analysis, related to Figures S1-10.

Movies S1-6.

Movie S1. Optogenetic activation of STN neurons in the dyskinesia state.

Movie S2. Optogenetic inactivation of STN neurons in the dyskinesia state.

Movie S3. Optogenetic activation of STN^{EP} neurons in the dyskinesia state.

Movie S4. Optogenetic activation of STN^{RtTg} neurons in the dyskinesia state.

Movie S5. Optogenetic inactivation of STN^{EP} neurons in the dyskinesia state.

Movie S6. Optogenetic inactivation of STN^{RtTg} neurons in the dyskinesia state.
